# Pathways explaining racial/ethnic and socio-economic disparities in dementia incidence: the UK Biobank study

**DOI:** 10.18632/aging.205058

**Published:** 2023-09-25

**Authors:** May A. Beydoun, Hind A. Beydoun, Marie T. Fanelli-Kuczmarski, Jordan Weiss, Michael F. Georgescu, Osorio Meirelles, Donald M. Lyall, Michele K. Evans, Alan B. Zonderman

**Affiliations:** 1 Laboratory of Epidemiology and Population Sciences, National Institute on Aging, NIA/NIH/IRP, Baltimore, MD 21224, USA; 2 Department of Research Programs, Fort Belvoir Community Hospital, Fort Belvoir, VA 22060, USA; 3 Stanford Center on Longevity, Stanford University, Stanford, CA 94305, USA; 4 School of Health and Wellbeing, University of Glasgow, Glasgow, Scottland, UK

**Keywords:** dementia, Alzheimer’s disease, health disparities, socio-economic status, structural equations modeling

## Abstract

Background: Pathways explaining racial/ethnic disparities in dementia risk are under-evaluated.

Methods: We examine those disparities and their related pathways among UK Biobank study respondents (50–74 y, *N* = 323,483; 3.6% non-White minorities) using a series of Cox proportional hazards and generalized structural equations models (GSEM).

Results: After ≤15 years, 5,491 all-cause dementia cases were diagnosed. Racial minority status (RACE_ETHN, Non-White vs. White) increased dementia risk by 24% (HR = 1.24, 95% CI: 1.07–1.45, *P* = 0.005), an association attenuated by socio-economic status (SES), (HR = 1.12, 95% CI: 0.96–1.31). Total race-dementia effect was mediated through both SES and Life’s Essential 8 lifestyle sub-score (LE8_LIFESTYLE_), combining diet, smoking, physical activity, and sleep factors. SES was inversely related to dementia risk (HR = 0.69, 95% CI: 0.67, 0.72, *P* < 0.001). Pathways explaining excess dementia risk among racial minorities included ‘RACE_ETHN(−) → SES(−) → DEMENTIA’, ‘RACE_ETHN(−) → SES(−) → Poor cognitive performance, COGN(+) → DEMENTIA’ and ‘RACE_ETHN(−) → SES(+) → LE8_LIFESTYLE_(−) → DEMENTIA’.

Conclusions: Pending future interventions, lifestyle factors including diet, smoking, physical activity, and sleep are crucial for reducing racial and socio-economic disparities in dementia.

## INTRODUCTION

Healthy cognitive functioning is required for performing activities of daily living, including attention, working or short-term memory, long-term memory, reasoning, movement coordination, and task-planning. The prevalence of brain disorders affecting cognition – such as stroke and dementia – increases with advancing age. Dementia is the loss of global abilities in multiple cognitive domains accompanied by the inability to perform usual activities of daily living dependence. The estimated prevalence of dementia is 4.7% among adults over 60 y [1], with 4.6–7.7 million cases added each year worldwide (3.5–10.5 per 1,000) [1–3]. Alzheimer’s disease (AD), the most common form of dementia, accounts for 60–80% of cases [1]. A progressive neurodegenerative disorder known for its multi-factorial etiology, AD manifests with episodic memory deterioration followed by impairment in other cognitive domains [4]. AD is likely caused by age-dependent and progressive Aβ-amyloid brain deposition, termed “the amyloid cascade hypothesis” [5]. AD is also characterized by neurofibrillary tangles (NFT), a second pathological hallmark that arises from hyper-phosphorylated tau protein [6]. AD is the leading cause of old age disability [7]. In developed countries, AD carries a greater health care burden.

With no current effective treatment, dementia prevention is crucial. Despite late-onset AD’s partial genetic basis (e.g. ApoE *ε4*), the 2020 *Lancet commission* reported that 40% of dementia’s risk can be attributed to early-life, mid-life and later life modifiable risk factors, including education, hearing loss, traumatic brain injury, hypertension, alcohol use obesity, smoking, depression, social isolation, physical inactivity, air pollution and diabetes [8]. Identification of novel mid-life risk factors and pathways between early and mid-life factors are thereby crucial in prevention efforts and for planning cost-effective interventions. Furthermore, cognitive decline and dementia have been positively associated with disadvantaged socioeconomic status (SES) [9, 10]. Socioeconomic status is commonly measured with education, occupation and income, with the former two being more relevant for dementia [11]. Neighborhood-level socioeconomic disadvantage including neighborhood structure, health outcomes within the area, personal housing and personal economics is also recognized as speeding cognitive decline in older adults [12]. Despite inconsistent evidence, long-term exposure to greenspace was associated with slower global cognition decline across the life span [13].

Among US adults, there are large racial disparities in numerous dementia risk factors, including obesity and related cardio-metabolic risk factors [14–16]. Moreover, wide racial, ethnic, and socio-economic disparities are found in AD and dementia incidence, with minority status and lower SES having adverse effects, often in combination. Related mediating pathways remain generally unexplored, particularly in the UK population [17–23].

The present study examines pathways that might explain racial, ethnic, and socio-economic disparities in AD or all-cause dementia in a large cohort study, the UK Biobank. Our study used several methodologies, including structural equation modeling coupled with survival analysis techniques to examine complex mediating effects between race, ethnicity, socioeconomic status, and dementia or AD risk in a sex-specific manner focusing on lifestyle, biological and cognitive pathways. It is also an attempt at replicating a previous study conducted among US older adults [24].

## MATERIALS AND METHODS

### Database

The UK Biobank is a prospective study of approximately 500,000 adults aged 37–73 y at baseline residing in the UK, and who were recruited between 2006 and 2010 [25]. Study rationale and design are detailed elsewhere [25]. Recruited participants attended one of 22 assessment centres (within 25 miles) in either England, Scotland, or Wales, completing a self-administered and touch-screen questionnaires as well as a face-to-face interview [25]. Phenotypic measurements and biological samples were collected [25]. After a careful review of former observational studies, clinical trials and population surveys and consulting with international experts, the UK Biobank questionnaire identified a wide array of quantifiable exposures in a wide range of interest areas [25].

### Standard protocol approvals, registrations, and patient consents

The study was approved by the North West Multi-Centre Research Ethics Committee, while participants provided written informed consent for data collection, data analysis, and record linkage, provided that the data was de-identified [25]. This analysis was approved by the UK Biobank access management team, as part of application #77963 and the project was approved by the Institutional Review Board of the National Institutes of Health.

### Incident AD and all-cause dementia

Focusing on the algorithmically derived dementia outcomes (fields 42018 and 42020), we excluded participants with onset of dementia occurring prior to baseline assessment [26]. The algorithm used included ICD-10 codes F00 or G30 for incident diagnosis for AD, whereas a number of codes were used for all-cause dementia, including vascular dementia (F01, I67.3), namely A81.0, F00, F01, F02, F03, F05, G30, G31.0, G31.1, G31.8, and I67.3. Date of the earliest occurrence of all-cause dementia was defined using the minimum of several date variables/fields that were available for each of the two outcomes [26].

### Race/ethnicity

Participants’ race/ethnicity was self-reported and was categorized in this study as White, Black, South Asian and Others as was done in a previous US study [24]. Moreover, the Non-White vs. White contrast was used in the main part of the pathway analyses. In our main analyses, RACE_ETHN referred to “racial minority status”, mainly contrasting Non-White to White (referent category).

### Mediators

#### Socio-economic status

Socio-economic status was operationalized with 3 different measures: education, income and Townsend deprivation index. Baseline self-reported completed education was recoded as follows: 0 = Low, combining None, “CSEs/Equivalent”, “NVQ/HND/HNC/Equivalent” and “Other professional qual”; 1 = Intermediate, combining “O Levels/GCSEs/Equivalent” and “A/AS Levels Equivalent; 2 = Higher level or “College/University” [27]. Total household income before tax was measured on a 5-point scale with 1 denoting less than £18,000, 2 £18,000–£29,999, 3 £30,000–£51,999, 4 £52,000–£100,000, and 5 greater than £100,000. The Townsend deprivation index (TDI) scores were computed based on national census data that measures residential postcode-level car ownership, household overcrowding, owner occupation, and unemployment. Originally coded to reflect higher socioeconomic deprivation with higher TDI scores [28], it was multiplied by -1 in this study in order to reflect higher SES and be combined with z-scores of education and income into one SES summary score.

### Study sample

Of the initial 502,399 UK Biobank participants, 384,627 were aged ≥50 y at baseline of whom 323,602 had available data on cognitive performance tests administered as well as all other key socio-demographic, SES, lifestyle and biological factors, including the LE8_LIFESTYLE_ and the LE_BIOLOGICAL_ scores. We additionally excluded 119 prevalent dementia cases at baseline assessment, which yielded a sample size of 323,483, of whom 2,314 had incident AD and 5,491 had incident all-cause dementia through the follow-up period of up to 15 years (Supplementary Figure 1). Participants who were excluded from analysis due to missing covariates differed from the remaining participants who were included, given an age at recruitment ≥50 y, by being younger, with lower likelihoods of being female or individuals from a racial minority group (*P* < 0.001), based on a multi-variable logistic regression model with selection (yes vs. no) as the outcome variable.

### Life’s essential 8

In 2010, the American Heart Association (AHA) defined a new measure of cardiovascular health (CVH) aiming at individual and population-level health promotion [29, 30]. CVH was initially operationalized with 7 potentially modifiable biological and lifestyle factors, that, when at optimal levels would result in greater cardiovascular disease (CVD)–free survival, longevity, and better quality of life. This measure was labelled “Life’s Simple 7” (LS7), with its 7 components: better diet quality, greater physical activity, reduced cigarette smoking, lower body mass index (BMI), total cholesterol, fasting blood glucose, and optimal blood pressure levels. Using clinical thresholds, each metric was categorized as poor (0), intermediate (1), or ideal (2), with total score range from 0 to 14 [29, 30]. Upon re-evaluation, a new measure labeled “Life’s Essential 8” (LE8) was formulated, retaining all 7 components of LS7 with major modifications to definitions and scales and by adding sleep health as an 8th component [30, 31] and one of four components of the LE8 lifestyle sub-scale. BMI, total cholesterol, glucose level, and blood pressure were included in the LE8_BIOLOGICAL_ sub-scale. Both sub-scales of LE8 (LE8_LIFESTYLE_ and LE8_BIOLOGICAL_) were tested as potential mediators in our present study, reflecting better CVH with higher score. Proration was applied to all potential mediators following the guidelines of <50% missing per scale [32] (See Supplementary Tables 1–4 and Supplementary Methods 1 and 2) [32].

### Lifestyle and health-related factors

In a supplementary analysis comparable to recent US studies [24, 33], lifestyle factors of interest included six concepts, namely “SMOKING”, “ALCOHOL”, “PHYSICAL ACTIVITY (PA)”, “DIET QUALITY (DIET)”, “NUTRITIONAL BIOMARKERS (NUTR)” and “SOCIAL SUPPORT (SS)”. The poor general and cardio-metabolic health construct (HEALTH) combined body mass index (BMI), the allostatic load (AL), a co-morbidity index and self-rated health. All these measures are detailed in Supplementary Methods 1–3 and Supplementary Table 5.

### Cognitive performance

Three cognitive test scores, available for most UK Biobank participants, included reaction time, pairs matching time to completion, and pairs matching number of errors. After being Log_e_ transformed, their z-scores were averaged to generate the COGN construct, reflecting poor cognitive performance in domains of visual memory and reaction time. The uni-dimensionality of COGN was tested using principal components analyses, from which the final COGN score was predicted (Supplementary Method 4).

### Exogenous covariates

Exogenous variables included age at baseline assessment, sex and household size. Moreover, sex was also considered as a key effect modifier in our analyses, while race/ethnicity was an exogenous variable in analyses with SES as the main exposure.

### Statistical methods

All analyses used Stata 17.0 (StataCorp, College Station, TX, USA), and were mostly stratified by sex. Comparison with race/ethnicity groups and sex as key predictors, used OLS linear, logistic and multinomial logit models, comparing means and proportions of variables of interest. Specifically, race/ethnicity was categorized as Non-White vs. White and sex differences were examined in the overall sample. Moreover, racial/ethnic differences in main characteristics were examined within each sex group.

We defined time-to-event (in years) from age at entry ≥50 y (i.e., delayed entry) until age of exit when event of interest or censoring (death or end of follow-up) would have occurred. AD and DEMENTIA incidence rates (IR, with 95% CI) were estimated across race/ethnicity groups by sex. In the main analysis, we conducted nested and sex-stratified Cox proportional hazards (PH) models on imputed data whereby socio-demographic, SES, lifestyle, health, and cognitive performance factors were entered consecutively in five models for both outcomes, while testing heterogeneity of race/ethnicity by sex by adding interaction terms to unstratified models. LE8 sub-scales were entered into models where SES and COGN were adjusted for.

**Figure 1:**
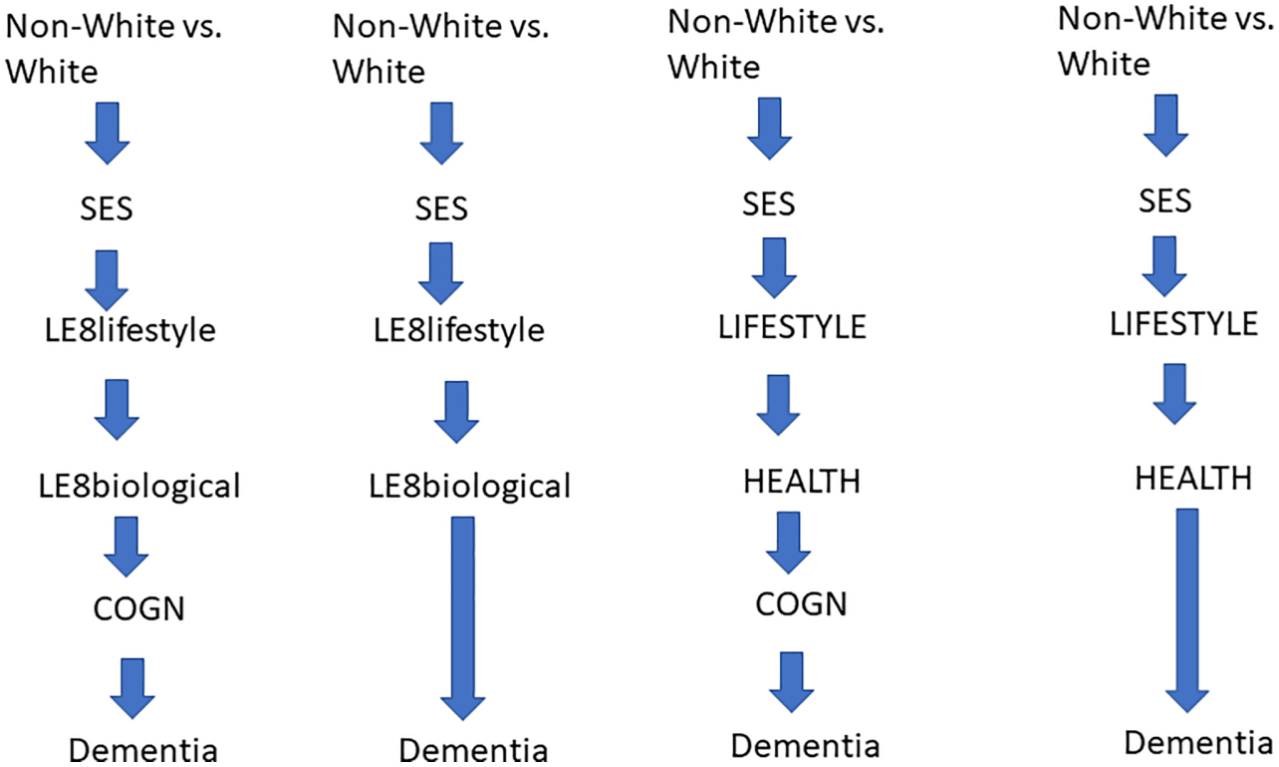
**Conceptual framework.** Abbreviations: ALCOHOL: Alcohol consumption *z*-score; COGN: Poor cognitive performance, *z*-score; DIET: Diet quality z-score; HEALTH: Poor cardio-metabolic and general health *z*-score; LE8_BIOLOGICAL_: Biological sub-scale of Life’s Essential 8; LE8_LIFESTYLE_: Lifestyle sub-scale of Life’s Essential 8; LIFESTYLE: Lifestyle factors including DIET, PA, SMOKING, ALCOHOL, NUTR and SS; NUTR: Nutritional biomarker *z*-score; PA: Physical Activity *z*-score; SES: Socio-economic status; SMOKING: Smoking *z*-score; SS: Social Support *z*-score.

Mediation was further examined using parametric survival models (Weibull GSEM), optimal for causal mediation in survival analysis [34]. Within GSEM, time to dementia (TD) was modeled as the outcome. GSEM models tested mediating pathways between Non-White vs. White contrast and the outcome. The main pathways dictate that SES z-score predicts LE8’s lifestyle component which predicts LE8’s biological component. The latter was allowed to predict “COGN” (higher z-score → poorer performance), which was hypothesized to directly influence AD or DEMENTIA risk. Importantly, other pathways were also allowed, including between endogenous variables and between RACE_ETHN and each endogenous variable (Figure 1). The total effects of RACE_ETHN and SES were estimated using GSEM where only exogenous variables were included with outcome being time to dementia incidence (Weibull model, Eq. 1). RACE_ETHN was included among exogenous variables in the model whereby SES total effect is to be estimated.

Direct effects in a structured manner represent the main pathway: direct effects into final TD, relationships between endogenous variables outside the pathway, and direct effects of race contrast outside the pathway. Indirect effects were also estimated by multiplying and adding effects from race/ethnicity into the final outcome, and passing through each mediator [35], including pathways from race/ethnicity to TD, through SES → LE8_LIFESTYLE_ → LE8_BIOLOGICAL_ → POOR COGNITIVE PERFORMANCE (COGN) → DEMENTIA, which was hypothesized to be the main pathway. Those models (Models A) included COGN as most proximal mediator to dementia outcome. In another set of models (Models B), COGN was omitted and effects of SES and LE8 sub-scales, among others, directly predicted TD. Exogenous variables were added to all equations. Furthermore, total effect of SES on AD/DEMENTIA was studied through similar pathways, the main hypothesized pathway being SES → LE8_LIFESTYLE_ → LE8_BIOLOGICAL_ → COGN → DEMENTIA. Finally, a sensitivity analysis, DIET, PA, SMOKING, NUTR and SS were included in the GSEM model with COGN as alternative lifestyle factors, while HEALTH was entered instead of LE8_BIOLOGICAL_. Direct and indirect effects are presented like the previous model with COGN. In all models, we adjusted for sample selectivity due to missing exposure and outcome data, relative to the initially recruited sample, using a two-stage Heckman selection strategy [36]. Initially, we predicted an indicator of selection with socio-demographic factors, namely, age, race/ethnicity and sex using probit regression, which yielded an inverse mills ratio (IMR) – a function of probability of being selected given those socio-demographic factors. Subsequently, we estimated our Cox proportional hazards regression and GSEM models adjusted for the IMR in addition to afore-mentioned covariates [36, 37], using 0.05 as Type-I error.

### Eq 1. Weibull distribution


$$f(x;\lambda ,k)=\frac{K}{\lambda }{\left(\frac{x}{\lambda }\right)}^{k-1}\;e-{\left(\frac{x}{\lambda }\right)}^{k}$$

Where λ is the scale parameter; *k* is the shape parameter; *x* is time to failure.

Y is f(*x*;*λ*,*k*) for *x* ≥ 0 and Y is 0 for *x* < 0.

### Data availability statement

The data analyzed in this study is subject to the following licenses/restrictions: UK Biobank is a large-scale biomedical database and research resource, containing in-depth genetic and health information from half a million United Kingdom participants. The database is regularly augmented with additional data and is globally accessible to approved researchers undertaking vital research into the most common and life-threatening diseases. Requests to access these datasets should be directed to https://www.ukbiobank.ac.uk/.

## RESULTS

The selected sample consisted of 323,483 adults, of whom 5,491 had incident all-cause dementia (2,314 were AD) through 15 years of follow-up. Table 1 and Supplementary Table 6 show study sample characteristics across key socio-demographics (sex and race), with results summarized in Supplementary Results 1. Most notably, SES *z*-score was significantly lower among racial minority groups, as were LE8 total and sub-scale scores.

**Table 1:** Study sample characteristics by race/ethnicity: The UK Biobank 2006–2021^a^.

**Study sample characteristics**	**All participants**	**Both sexes combined, *n* = 323,483**		***P***
		**White**	**Non-white**	
**Socio-demographic**				
** Baseline age, y**	60.4 ± 5.4	60.5 ± 5.4	58.6 ± 5.6	<0.001
** Sex, % female**	53.6	53.6	53.8	0.81
** Race/ethnicity**				—
White	96.4	100.0	0.0	—
Black	0.9	0.0	25.2	—
South Asian	1.2	0.0	33.6	—
Other	1.5	0.0	41.1	—
** Household size**	2.2 ± 1.2	2.2 ± 1.1	2.7 ± 1.6	<0.001
**Socio-economic status**				
** Education**				
Low	21.8	21.7	23.4	—
Intermediate	39.6	40.0	29.8	<0.001
High	38.6	38.3	46.8	<0.001
** Income**				<0.001
Less than £18,000	25.3	25.2	30.3	—
£18,000–£29,999	28.0	28.0	27.8	—
£30,000–£51,999	24.9	24.9	22.5	—
£52,000–£100,000	17.4	17.5	15.1	—
greater than £100,000	4.4	4.4	4.3	—
** TDI**	−1.56 ± 2.95	−1.63 ± 2.90	0.47 ± 3.48	<0.001
** SES z-score**	−0.03 ± 0.70	−0.02 ± 0.70	−0.28 ± 0.79	<0.001
**Lifestyle factors**				
**Smoking**				
** Smoking status**				
Never	81.3	81.2	86.0	—
Former	9.5	9.7	4.2	<0.001
Current	9.2	9.2	9.9	0.61
Environmental tobacco smoke	0.88 ± 5.2	0.88 ± 5.26	1.03 ± 4.72	0.002
Pack-years of tobacco smoke	0.08 ± 0.26	0.08 ± 0.26	0.05 ± 0.19	<0.001
** SMOKING z-score**	−0.005 ± 0.442	−0.004 ± 0.442	−0.025 ± 0.418	<0.001
**Alcohol consumption**				
** Alcohol consumption frequency**				
0 "never"	7.3	6.6	24.5	—
1 "special occasions only"	11.1	10.6	23.7	<0.001
2 "1–3 times per month"	10.4	10.4	10.9	<0.001
3 "1–3 times per week"	24.7	25.0	18.2	<0.001
4 "3–4 times per week"	23.7	24.2	12.0	<0.001
5 "daily or almost daily"	22.8	23.2	10.7	<0.001
**ALCOHOL z-score**	0.00 ± 1.00	+0.03 ± 0.98	−0.743 ± 1.11	<0.001
**Physical activity, PA**				
** PA, Met.min.wk^-1^**	1,963 ± 2,812	1,971 ± 2,817	1,772 ± 2,796	<0.001
** PA z-score**	0.00 ± 1.00	+0.00 ± 1.00	−0.068 ± 0.992	<0.001
**Diet quality**				
** HDI total score**	5.11 ± 1.50	5.10 ± 1.50	5.36 ± 1.43	<0.001
** DIET z-score**	0.00 ± 1.00	−0.01 ± 1.00	0.17 ± 0.96	<0.001
**Nutritional Biomarkers**				
** 25-hydroxyvitamin D**	49.6 ± 20.9	50.2 ± 20.8	35.4 ± 18.1	<0.001
** Red cell distribution width**	13.5 ± 0.9	13.5 ± 0.9	13.8 ± 1.2	<0.001
** NUTR z-score**	−0.001 ± 0.757	+0.017 ± 0.746	−0.496 ± 0.871	<0.001
**Social Support**				
** “How often do you visit friends or family or have them visit you?”**	5.27 ± 1.13	5.28 ± 1.13	4.84 ± 1.21	<0.001
** “How often are you able to confide in someone close to you?”**	1.04 ± 0.87	1.04 ± 0.87	0.95 ± 0.83	<0.001
** “Which of the following do you attend once a week or more often?”**	3.55 ± 1.89	3.56 ± 1.88	3.03 ± 1.98	<0.001
** SS z-score**	−0.001 ± 0.630	+0.008 ± 0.631	−0.254 ± 0.669	<0.001
**Cardio-metabolic and general health-related factors**				
**Body mass index, kg.m^-1^**	27.5 ± 4.7	27.5 ± 4.7	27.8 ± 5.0	<0.001
**Allostatic load**	2.10 ± 1.39	2.10 ± 1.38	2.23 ± 1.41	<0.001
**Co-morbidity index**	2.11 ± 1.94	2.11 ± 1.94	2.12 ± 1.92	<0.001
**Self-rated health**				<0.001
** Excellent**	16.5	16.7	11.2	
** Good**	59.0	59.2	53.2	
** Fair**	20.4	20.1	28.8	
** Poor**	4.1	4.0	6.7	
**HEALTH z-score**	0.0004 ± 0.687	−0.004 ± 0.687	0.110 ± 0.701	<0.001
**Cognitive performance**				
** Reaction Time**	6.33 ± 0.19	6.32 ± 0.18	6.41 ± 0.22	<0.001
** Pairs matching, errors**	0.72 ± 0.70	0.70 ± 0.70	0.99 ± 0.73	<0.001
** Pairs matching, time to complete**	5.35 ± 0.37	5.34 ± 0.4	5.57 ± 0.46	<0.001
** COGN z-score**	0.000 ± 0.756	−0.018 ± 0.743	0.481 ± 0.917	<0.001
**LE8**				
** Total score**	502.3 ± 95.6	502.8 ± 95.6	488.8 ± 95.2	<0.001
** Biological score**	246.4 ± 65.9	246.8 ± 65.7	236.0 ± 63.1	<0.001
** Lifestyle score**	255.9 ± 63.3	256.0 ± 63.3	251.9 ± 63.1	<0.001
**Incidence proportion**				
** All-cause dementia**	1.70 (*n* = 5,491)	1.71 (*n* = 5,321)	1.45 (*n* = 170)	0.035
** AD dementia**	0.72 (*n* = 2,314)	0.72 (*n* = 2,245)	0.59 (*n* = 69)	0.098

Abbreviations: AD: Alzheimer’s Disease; ALCOHOL: Alcohol consumption z-score; COGN: Poor cognitive performance z-score; DIET: diet quality z-score; HDI: Healthy Diet Index; HEALTH: Poor cardio-metabolic and general health z-score; LE8: Life’s Essential 8; PA: Physical Activity z-score; NUTR: Nutritional biomarker z-score; SES: Socio-economic status z-score; SMOKING: Smoking z-score; SS: Social Support z-score; TDI: Townsend Deprivation Index. ^a^Values are percentages or means +/− standard deviations. *P* for null hypothesis of no difference by race.

Table 2 presents Cox proportional hazards model findings, focusing on racial/ethnic disparities in incident all-cause and AD dementia. Adjusted for only exogenous variables (age, household size, and sex for non-stratified models), Model 1 shows that Black adults had on average 1.8 to 2.2-fold risk of all-cause dementia compared to their White counterparts in both sexes. This ethnic and racial disparity was markedly attenuated when SES was entered into the model (Model 2), particularly among women. Among men, the HR was non-significant upon further adjustment for lifestyle factors (Model 3). A similar gap was found for AD dementia outcome. In contrast, no disparity was detected between South Asian and White adults, and this contrast was inversely related to all-cause dementia incidence upon adjustment for baseline cognitive performance in both sexes (Model 5: women HR = 0.56, 95% CI: 0.34–0.9; men HR = 0.76, 95% CI: 0.62–0.94). Overall, non-White adults, particularly men, were at 24% greater risk for all-cause dementia compared to their White counterparts in Model 1. This association was attenuated after entering SES into the model (HR = 1.12, 95%CI: 0.96–1.31) and inverted when all lifestyle, health-related and cognitive performance scores were included (Model 5: HR = 0.75, 95% CI: 0.64–0.87). For AD, there was no disparity detected in Model 1, which then became an inverse relationship of Non-White vs. White with AD incidence in fully adjusted model 5. Nevertheless, in Model 6, which included cognitive performance, SES, LE8 sub-scores and exogenous variables, no relationship between race/ethnicity (Non-White vs. White) and dementia outcomes was detected.

**Table 2:** Racial/ethnic disparities in incident all-cause and Alzheimer’s disease dementia among middle-aged males and females (*N* = 323,483): Cox proportional hazards models; The UK Biobank 2006–2021^a^.

	**All-cause Dementia**			**AD Dementia**		
	**HR**	**(95% CI)**	***P***	**HR**	**95% CI**	***P***
**Males, Black vs. White**						
Model 1	**2.18**	**(1.53, 3.11)**	**<0.001**	**1.87**	**(1.00, 3.48)**	**0.049**
Model 2	**1.63**	**(1.14, 2.33)**	**0.007**	1.45	(0.78, 2.72)	0.24
Model 3	1.32	(0.92, 1.88)	0.13	1.28	(0.68, 2.40)	0.44
Model 4	1.41	(0.99, 2.02)	0.058	1.31	(0.70, 2.46)	0.40
Model 5	0.96	(0.67, 1.37)	0.82	0.89	(0.47, 1.68)	0.72
Model 6	1.06	(0.74, 1.52)	0.75	0.96	(0.51, 1.81)	0.90
**Females, Black vs. White**						
Model 1	**1.83**	**(1.25, 2.67)**	**0.002**	**1.92**	**(1.11, 3.32)**	**0.019**
Model 2	1.41	(0.96, 2.07)	0.079	1.46	(0.84, 2.54)	0.18
Model 3	1.17	(0.80, 1.73)	0.41	1.23	(0.71, 2.15)	0.46
Model 4	1.12	(0.76, 1.65)	0.55	1.19	(0.68, 2.07)	0.54
Model 5	0.77	(0.53, 1.14)	0.20	0.80	(0.46, 1.39)	0.43
Model 6	0.95	(0.66, 1.40)	0.80	0.93	(0.53, 1.63)	0.81
**Males, South Asian vs. White**						
Model 1	1.06	(0.75, 1.48)	0.76	1.18	(0.71, 1.97)	0.53
Model 2	1.00	(0.71, 1.40)	0.98	1.13	(0.68, 1.89)	0.63
Model 3	0.77	(0.54, 1.08)	0.13	0.97	(0.58, 1.64)	0.92
Model 4	0.79	(0.56, 1.11)	0.17	0.98	(0.59, 1.65)	0.95
Model 5	**0.60**	**(0.42, 0.85)**	**0.004**	0.76	(0.45, 1.27)	0.29
Model 6	0.75	(0.53, 1.05)	0.093	0.85	(0.51, 1.42)	0.54
**Females, South Asian vs. White**						
Model 1	0.95	(0.58, 1.56)	0.85	1.13	(0.59, 2.18)	0.71
Model 2	0.91	(0.56, 1.49)	0.71	1.09	(0.57, 2.11)	0.79
Model 3	0.72	(0.44, 1.18)	0.20	0.90	(0.46, 1.74)	0.75
Model 4	0.73	(0.44, 1.19)	0.21	0.90	(0.47, 1.75)	0.76
Model 5	**0.56**	**(0.34, 0.92)**	**0.022**	0.68	(0.35, 1.33)	0.26
Model 6	0.70	(0.43, 1.15)	0.16	0.81	(0.42, 1.56)	0.52
**Males, Non-White vs. White**						
Model 1	**1.26**	**(1.03, 1.54)**	**0.025**	1.20	(0.86, 1.68)	0.28
Model 2	1.14	(0.93, 1.40)	0.20	1.11	(0.79, 1.55)	0.54
Model 3	0.93	(0.76, 1.15)	0.52	0.99	(0.70, 1.38)	0.94
Model 4	0.96	(0.78, 1.18)	0.73	1.00	(0.71, 1.40)	0.98
Model 5	**0.76**	**(0.62, 0.94)**	**0.010**	0.80	(0.57, 1.12)	0.19
Model 6	0.88	(0.72, 1.08)	0.24	0.87	(0.62, 1.22)	0.42
**Females, Non-White vs. White**						
Model 1	1.23	(0.97, 1.56)	0.084	1.22	(0.86, 1.72)	0.27
Model 2	1.11	(0.87, 1.40)	0.40	1.09	(0.77, 1.55)	0.61
Model 3	0.93	(0.73, 1.18)	0.55	0.94	(0.66, 1.34)	0.74
Model 4	0.93	(0.73, 1.18)	0.55	0.94	(0.66, 1.34)	0.73
Model 5	**0.73**	**(0.57, 0.93)**	**0.010**	0.72	(0.51, 1.03)	0.076
Model 6	0.86	(0.68, 1.09)	0.22	0.82	(0.58, 1.17)	0.28
**Overall, Non-White vs. White**						
Model 1	**1.24**	**(1.07, 1.45)**	**0.005**	1.20	(0.95, 1.53)	0.13
Model 2	1.12	(0.96, 1.31)	0.14	1.10	(0.86, 1.40)	0.44
Model 3	0.93	(0.80, 1.09)	0.38	0.96	(0.75, 1.23)	0.75
Model 4	0.95	(0.81, 1.11)	0.51	0.97	(0.76, 1.24)	0.79
Model 5	**0.75**	**(0.64, 0.87)**	**<0.001**	**0.76**	**(0.59, 0.97)**	**0.030**
Model 6	0.88	(0.76, 1.03)	0.12	0.85	(0.69, 1.09)	0.20

Abbreviations: AD: Alzheimer’s Disease; ALCOHOL: Alcohol consumption z-score; COGN: Poor cognitive performance z-score; DIET: diet quality z-score; HEALTH: Poor cardio-metabolic and general health z-score; NUTR: Nutritional biomarker z-score; PA: Physical Activity z-score; RACE_ETHN: Race/ethnicity; SES: Socio-economic status z-score; SMOKING: Smoking z-score; SS: Social Support z-score. ^a^Values are β ± SE (Log_e_(HR)). Model 1: adjusted for age (or age and sex); Model 2: adjusted for demographic factors other than age and sex, and SES score; Model 3: Model 2 further adjusted for lifestyle-related factors (average of z-scores of measured variables for SMOKING, ALCOHOL, DIET, NUTR, SS and PA); Model 4: Model 3 + health-related factors (HEALTH score); Model 5: Full model with cognitive test PCA score; Model 6: is Model 2+LE8 lifestyle and biological sub-scales+ cognitive test PCA score. *P* for null hypothesis that Loge(HR) = 0.

Table 3 focuses on potential socio-economic disparities in all-cause and AD dementia after adjusting for race and ethnicity (Non-White vs. White) and other exogenous variables. SES was a statistically significant predictor for dementia risk, even upon adjustment for lifestyle, health-related and cognitive performance factors. LE8_LIFESTYLE_ was an independent predictor for reduced all-cause dementia risk, independently from LE8_BIOLOGICAL_, SES, race and ethnicity, and baseline cognitive performance. However, neither LE8_LIFESTYLE_ nor LE8_BIOLOGICAL_ were associated with AD; only lower SES and poor cognitive performance were important predictors. In Model 4, the HEALTH construct, reflecting poor cardiometabolic and general health, directly predicted both AD and all-cause dementia, while greater social support, alcohol consumption and higher levels of nutritional biomarkers were among lifestyle factors that were inversely related to dementia and AD risk, independently of SES and baseline cognitive performance.

**Table 3:** Socio-economic disparities in incident all-cause and Alzheimer’s disease dementia among middle-aged adults (*N* = 323,483): Cox proportional hazards models; The UK Biobank 2006–2021^a,b^.

	**All-cause dementia**			**AD dementia**		
	**HR**	**95% CI**	***P***	**HR**	**95% CI**	***P***
**Model 1**						
Non-White vs. White	1.12	(0.96, 1.31)	0.14	1.10	(0.86, 1.40)	0.44
SES	**0.69**	**(0.67, 0.72)**	**<0.001**	**0.71**	**(0.66, 0.75)**	**<0.001**
**Model 2**						
Non-White vs. White	0.93	(0.80, 1.09)	0.38	0.96	(0.75, 1.23)	0.75
SES	**0.74**	**(0.71, 0.77)**	**<0.001**	**0.72**	**(0.68, 0.77)**	**<0.001**
SMOKING	0.98	(0.96, 1.02)	0.38	**0.89**	**(0.83, 0.95)**	**0.001**
DIET	1.01	(0.98, 1.03)	0.66	1.04	(1.00, 1.08)	0.070
PA	0.99	(0.96, 1.02)	0.68	**1.04**	**(1.01, 1.08)**	**0.024**
ALCOHOL	**0.90**	**(0.88, 0.93)**	**<0.001**	**0.92**	**(0.88, 0.97)**	**0.001**
NUTR	**0.85**	**(0.83, 0.88)**	**<0.001**	**0.91**	**(0.87, 0.96)**	**<0.001**
SS	**0.83**	**(0.80, 0.87)**	**<0.001**	**0.90**	**(0.84, 0.96)**	**0.001**
**Model 3**						
Non-White vs. White	0.95	(0.82, 1.11)	0.51	0.97	(0.76, 1.24)	0.79
SES	**0.78**	**(0.75, 0.82)**	**<0.001**	**0.75**	**(0.70, 0.80)**	**<0.001**
SMOKING	0.98	(0.94, 1.02)	0.26	**0.88**	**(0.83, 0.95)**	**<0.001**
DIET	**1.03**	**(1.00, 1.06)**	**0.026**	**1.05**	**(1.01, 1.10)**	**0.013**
PA	1.03	(1.04, 1.06)	0.054	**1.06**	**(1.02, 1.10)**	**0.002**
ALCOHOL	**0.93**	**(0.91, 0.96)**	**<0.001**	**0.94**	**(0.90, 0.98)**	**0.004**
NUTR	**0.90**	**(0.87, 0.93)**	**<0.001**	**0.94**	**(0.89, 1.00)**	**0.037**
SS	**0.84**	**(0.81, 0.88)**	**<0.001**	**0.90**	**(0.84, 0.96)**	**0.002**
HEALTH	**1.44**	**(1.38, 1.50)**	**<0.001**	**1.24**	**(1.16, 1.32)**	**<0.001**
**Model 4**						
Non-White vs. White	**0.75**	**(0.64, 0.87)**	**<0.001**	**0.76**	**(0.59, 0.97)**	**0.030**
SES	**0.83**	**(0.80, 0.87)**	**<0.001**	**0.79**	**(0.75, 0.85)**	**<0.001**
SMOKING	0.99	(0.95, 1.03)	0.60	**0.90**	**(0.84, 0.96)**	**0.002**
DIET	1.03	(1.00, 1.05)	0.06	**1.05**	**(1.01, 1.09)**	**0.025**
PA	1.02	(0.99, 1.04)	0.16	**1.05**	**(1.01, 1.09)**	**0.006**
ALCOHOL	**0.94**	**(0.92, 0.97)**	**<0.001**	**0.95**	**(0.91, 0.99)**	**0.023**
NUTR	**0.90**	**(0.87, 0.93)**	**<0.001**	0.95	(0.90, 1.00)	0.054
SS	**0.86**	**(0.82, 0.90)**	**<0.001**	**0.92**	**(0.86, 0.98)**	**0.010**
HEALTH	**1.44**	**(1.39, 1.50)**	**<0.001**	**1.25**	**(1.17, 1.32)**	**<0.001**
COGN	**1.50**	**(1.45, 1.55)**	**<0.001**	**1.50**	**(1.43, 1.58)**	**<0.001**
**Model 5**						
Non-White vs. White	0.88	(0.76, 1.03)	0.12	0.85	(0.67, 1.09)	0.20
SES	**0.77**	**(0.74, 0.80)**	**<0.001**	**0.76**	**(0.96, 1.04)**	**<0.001**
LE8_LIFESTYLE_	**0.88**	**(0.86, 0.90)**	**<0.001**	0.99	(0.95, 1.03)	0.77
LE8_BIOLOGICAL_	0.98	(0.96, 1.01)	0.21	0.99	(0.95, 1.02)	0.53
COGN	**1.50**	**(1.45, 1.55)**	**<0.001**	**1.51**	**(1.44, 1.60)**	**<0.001**

Abbreviations: AD: Alzheimer’s Disease; ALCOHOL: Alcohol consumption z-score; COGN: Poor cognitive performance z-score; DIET: diet quality z-score; HEALTH: Poor cardio-metabolic and general health z-score; LE8: Life’s Essential 8; NUTR: Nutritional biomarker z-score; PA: Physical Activity z-score; SES: Socio-economic status z-score; SMOKING: Smoking z-score; SS: Social Support z-score. ^a^Values are β ± SE (Log_e_(HR)). Model 1: adjusted for age, sex and race/ethnicity; Model 2: adjusted for demographic factors other than age, sex and race/ethnicity; Model 3: Model 2 further adjusted for lifestyle-related factors (average of z-scores of measured variables for SMOKING, ALCOHOL, DIET, NUTR, SS and PA); Model 4: Model 3 + health-related factors (HEALTH score); Model 5: Full model with cognitive test PCA score; Model 6: is Model 2+LE8 lifestyle and biological sub-scales+ cognitive test PCA score. ^b^*P* < 0.05 for sex × SES interaction in unstratified model. *P* for null hypothesis of Log_e_(HR) = 0.

Table 4 tests mediating effects in a more structured manner with LE8 sub-scores used among mediators by applying the GSEM approach. The results indicate that there was no direct association between Non-White vs. White contrast and dementia risk. However, several mediating pathways were uncovered, including SES as the key paths, particularly ‘RACE_ETHN(−) → SES(−) → DEMENTIA’ and ‘RACE_ETHN(−) → SES(−) → COGN(+) → DEMENTIA’. These paths accounted for approximately half of the total effect of race/ethnicity on dementia risk. In contrast, only 5% of the total effect was accounted for by ‘RACE_ETHN(−) → SES(+) → LE8_LIFESTYLE_(−) → DEMENTIA’.

**Table 4:** Total, direct, and indirect effects of race/ethnicity (Non-White vs. White) vs. time to all-cause dementia through SES, lifestyle, health-related and cognitive performance factors among middle-aged adults (Age_base_: 50–74 y); The UK Biobank 2006–2021^a^.

	**MODEL A**		**MODEL B**	
	**β**	**(SE), *p***	**β**	**(SE), *p***
*Main pathway*				
RACE_ETHN → SES (β_12_)	**−0.350**	**(0.006), <0.001**	**−0.351**	**(0.006), <0.001**
SES → LE8_LIFESTYLE_ (β_23_)	**+0.270**	**(0.003), <0.001**	**+0.270**	**(0.003), <0.001**
LE8_LIFESTYLE_ → LE8_BIOLOGICAL_ (β_34_)	**+0.098**	**(0.002), <0.001**	**+0.098**	**(0.002), <0.001**
LE8_BIOLOGICAL_ → COGN(β_45_)	**+0.025**	**(0.001), <0.001**	**—**	**—**
COGN → DEMENTIA (β_56_)	**+0.417**	**(0.018), <0.001**	**—**	**—**
*Selected direct effects on final outcomes*				
RACE_ETHN → DEMENTIA(β_16_)	−0.123	(0.079), 0.12	+0.133	(0.078), 0.089
SES → DEMENTIA(β_26_)	**−0.271**	**(0.020), <0.001**	**−0.338**	**(0.020), <0.001**
LE8_LIFESTYLE_ → DEMENTIA(β_36_)	**−0.113**	**(0.014), <0.001**	**−0.110**	**(0.014), <0.001**
LE8_BIOLOGICAL_ → DEMENTIA(β_46_)	−0.027	(0.014), 0.054	−0.017	(0.014), 0.24
*Other effects between endogenous variables*				
SES → LE8_BIOLOGICAL_ (β_24_)	**+0.122**	**(0.003), <0.001**	**+0.122**	**(0.003), <0.001**
SES → COGN (β_25_)	**−0.138**	**(0.002), <0.001**	**—**	**—**
LE8_LIFESTYLE_ → COGN (β_35_)	**+0.008**	**(0.001), <0.001**	**—**	**—**
*Other direct effects of race*				
RACE_ETHN → LE8_LIFESTYLE_ (β_13_)	**+0.040**	**(0.009), <0.001**	**+0.040**	**(0.009), <0.001**
RACE_ETHN → LE8_BIOLOGICAL_(β_14_)	**−0.162**	**(0.009), <0.001**	**−0.163**	**(0.009), <0.001**
RACE_ETHN → COGN(β_15_)	**+0.530**	**(0.006), <0.001**	**—**	**—**
*Selected Indirect effects*				
RACE_ETHN → SES → DEMENTIA(β_A_)	**+0.095**	**(0.007), <0.001**	**+0.119**	**(0.007), <0.001**
RACE_ETHN → SES → LE8_LIFESTYLE_ → DEMENTIA(β_B_)	**+0.011**	**(0.001), <0.001**	**+0.0104**	**(0.0013), <0.001**
RACE_ETHN → SES → LE8_LIFESTYLE_ → LE8_BIOLOGICAL_ → DEMENTIA(β_C_)	+0.0002	(0.0001), 0.054	+0.00015	(0.0001), 0.24
RACE_ETHN → SES → LE8_LIFESTYLE_ → LE8_BIOLOGICAL_ → COGN → DEMENTIA(β_D_)	**−0.00010**	**(0.0000), 0.001**	—	—
RACE_ETHN → SES → LE8_LIFESTYLE_ → COGN → DEMENTIA(β_E_)	**−0.00032**	**(0.00005), <0.001**	—	—
RACE_ETHN → SES → COGN → DEMENTIA(β_F_)	**+0.0202**	**(0.010), <0.001**	—	—
TOTAL EFFECT OF RACE_ETHN	**+0.232**	**(0.078), 0.003**	**+0.232**	**(0.078), 0.003**

Abbreviations: COGN: Poor cognitive performance z-score; DEMENTIA: Dementia; LE8: Life’s essential 8’; RACE_ETHN: Racial minority status (Non-White vs. White); SES: Socio-economic status z-score. ^a^Values are path coefficients β ± SE or non-linear combinations of path coefficients to compute selected indirect effects. → DEMENTIA associations are interpreted as Log_e_(HR) of these incident outcomes per unit exposure, as are total effects of RACE_ETHN. *P* for null hypothesis of β = 0.

**Figure 2:**
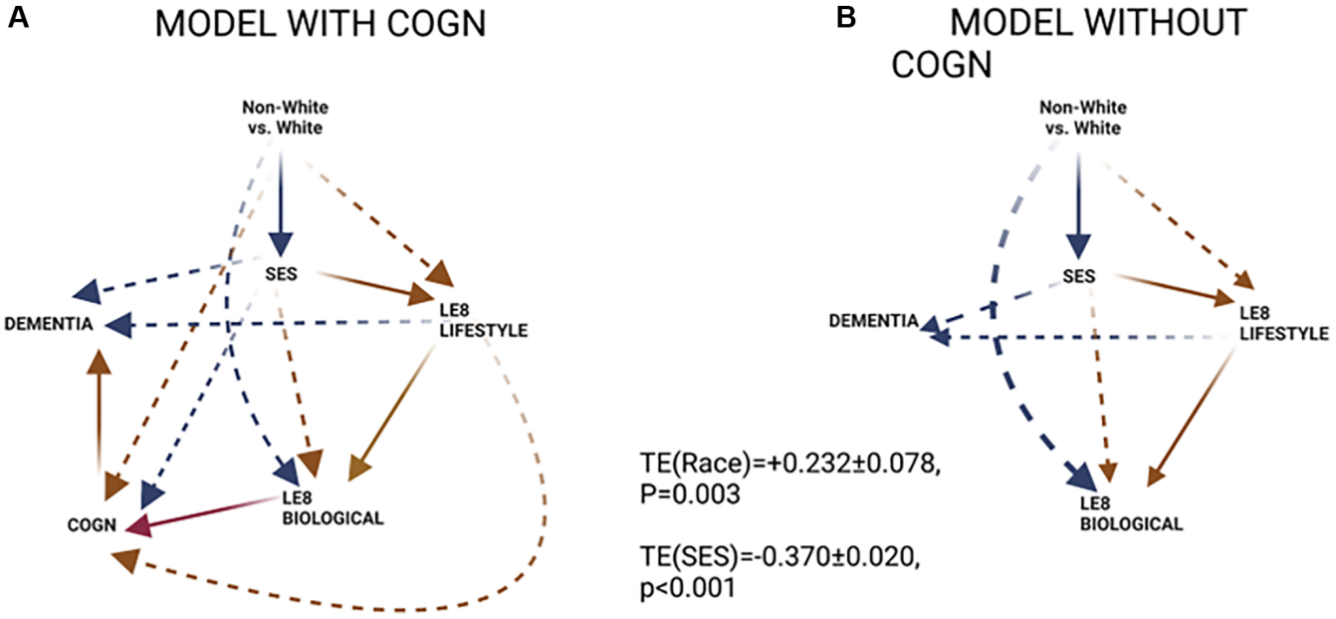
**GSEM findings.** (**A**) Model with COGN as a proximal mediator; (**B**) Model without COGN as a proximal mediator. Abbreviations: COGN: Poor cognitive performance, *z*-score; LE8_BIOLOGICAL_: Biological sub-scale of Life’s Essential 8; LE8_LIFESTYLE_: Lifestyle sub-scale of Life’s Essential 8; SES: Socio-economic status; Red lines: positive associations; Blue lines: inverse associations; Solid line: within hypothesized pathway; Dashed line: outside hypothesized pathway.

Table 5 examined the mediating roles of LE8 sub-scores and COGN in dementia risk’s socio-economic disparities using a similar GSEM approach and adjusting for exogenous variables in all equations. While the total effect of SES was an inverse one (TE = −0.370, *P* < 0.001), around 8% of this effect was explained by LE8_LIFESTYLE_ in comparison to 16% being explained by greater baseline cognitive performance. Thus, a large portion of the total effect SES on dementia risk was a direct effect, unexplained by the pathways under consideration. Findings from Tables 4 and 5 are illustrated further in a qualitative manner in Figure 2. Supplementary analyses using different potential mediators as shown in Figure 1, are presented in Supplementary Tables 7 and 8 and illustrated in Supplementary Figure 2 for Models A and B. Supplementary Results 1 summarizes the key findings. Most notably, social support and nutritional biomarkers were among mediators explaining a large portion of racial/ethnic disparities in dementia (17–25% of the total effect), without necessarily going through SES as an antecedent mediator. Nevertheless, in these models, SES mediated about half of the total race-dementia effect. It is also worth noting that in Model A, pathways going through COGN indicated possible reverse causation, with poor cognitive performance positively predicting dementia risk while being concurrently associated with improved dietary and other lifestyle habits. This pattern was also observed in Model A, Figure 2.

**Table 5:** Total and selected indirect effects of socio-economic status vs. all-cause dementia through LE8_LIFESTYLE_, LE8_BIOLOGICAL_ and cognitive performance factors among middle-aged adults (Age_base_: 50–74 y); The UK Biobank 2006–2021^a^.

	**MODEL A**		**MODEL B**	
	**β**	**(SE), *p***	**β**	**(SE), *p***
*Selected Indirect effects*				
SES → LE8_LIFESTYLE_ → DEMENTIA(β_A’_)	**−0.030**	**(0.004), <0.001**	**−0.030**	**(0.004), <0.001**
SES → LE8_LIFESTYLE_ → LE8_BIOLOGICAL_ → DEMENTIA(β_B’_)	−0.0007	(0.0004), 0.054	−0.0004	(0.0004), 0.24
SES → LE8_LIFESTYLE_ → LE8_BIOLOGICAL_ → COGN → DEMENTIA(β_C’_)	**+0.00027**	**(0.00002), <0.001**	—	—
SES → LE8_LIFESTYLE_ → COGN → DEMENTIA(β_D’_)	**+0.00092**	**(0.0002), <0.001**	—	—
SES → COGN → DEMENTIA(β_E’_)	**−0.0576**	**(0.0025), <0.001**	—	—
TOTAL EFFECT OF SES	**−0.370**	**(0.020), <0.001**	**−0.370**	**(0.020), <0.001**

Abbreviations: COGN: Poor cognitive performance z-score; DEMENTIA: Dementia; LE8: Life’s Essential 8; SES: Socio-economic status z-score. ^a^Values are path coefficients β ± SE or non-linear combinations of path coefficients to compute selected indirect effects → DEMENTIA associations are interpreted as Log_e_(HR) of these incident outcomes per unit exposure, as are total effect of SES. *P* for null hypothesis of β = 0.

## DISCUSSION

The present study is among few to examine racial/ethnic disparities in dementia risk and their related pathways among UK Biobank study respondents (50–74 y, *N* = 323,483; 3.6% non-White minorities) using a series of Cox proportional hazards and generalized structural equations models (GSEM). It is the first to do so in a UK population. Among key findings, and after ≤15 years, 5,491 all-cause dementia cases were diagnosed. Racial minority status increased dementia risk by 24% (HR = 1.24, 95% CI: 1.07–1.45, *P* = 0.005), an association attenuated by socio-economic status (SES), (HR = 1.12, 95% CI: 0.96–1.31). Total race-dementia effect was mediated through both SES and lifestyle factors (e.g., LE8_LIFESTYLE_). SES was inversely related to dementia risk (HR = 0.69, 95% CI: 0.67, 0.72, *P* < 0.001). Pathways explaining excess dementia risk among racial minorities included ‘RACE_ETHN(−) → SES(−) → DEMENTIA’, ‘RACE_ETH(−) → SES(−) → COGN(+) → DEMENTIA’ and ‘RACE_ETHN(−) → SES(+) → LE8_LIFESTYLE_(−) → DEMENTIA’.

Previous studies report lower SES to be associated with higher health risk behavior levels and generally reduced access to quality resources [38]. The latter is among key structural determinant that can link low SES to dementia occurrence, particularly among racial minority groups that have been historically marginalized [39]. Additive chronic stress triggered by low SES coupled with lack of social support can lead to an accumulation of allostatic load, a mechanism thought to explain the relationship between chronic stress and cognitive dysfunction [40]. Thus, lack of social support is an antecedent factor to cardiometabolic health, as is the case for socio-economic status, and can indirectly lead to adverse cognitive outcomes through factors such as allostatic load [39]. Additionally, a combination of low SES and chronic stress may trigger maladaptive responses leading to neuroendocrine, autonomic, and behavioral modifications, which are thought to directly related with poor cognitive function. For instance, the prefrontal cortex was shown to be negatively affected by chronic stress resulting from lower SES [41]. Thus, low SES is linked to a complex interplay of biological, physiological, and environmental factors which, in turn, results in cognitive dysfunction.

We found that SES is a key mediator between race and dementia incidence, and that it was sufficient in its mediating effect even though lifestyle and health-related factors as well as cognitive performance at baseline assessment had an important role to play in the race-dementia relationship. Previous studies suggest that there are marked racial disparities in occurrence of AD and related dementias [17, 18, 24]. In a multi-ethnic cohort, for instance, the age standardized diagnostic incidence rate of dementia from all causes was increased in African American (22.9 in women, 21.5 in men) and Native Hawaiian (19.3, 19.4) older adults compared to their White counterparts (16.4, 15.5), while being comparable in the Latino group (16.8, 14.7) and significantly reduced among Japanese American (14.8, 13.8), and Filipino (12.5, 9.7) older adults [18]. In another more recent study, incident all-cause dementia among older adults in the US was significantly greater among NHB women compared to NHW women, whereas Mexican-American women were at reduced AD risk compared with their NHW counterparts, especially upon further adjustment for SES and upstream factors [24]. SES mediated a large portion of the NHB-NHW women disparity in dementia, in addition to several other lifestyle factors, most notably diet and physical activity [24]. Income-level differences in pathways between race/ethnicity and dementia risk were observed in another comparable study, highlighting the importance of social support in reducing dementia risk within the lowest income category [33]. The socio-economic gradient in dementia incidence playing a major role in racial/ethnic disparities in this health outcome was also suggested in other studies [20–23]. More recently, beneficial effects ascribed to education included reduced cognitive adverse effects of tau accumulation, one of two hallmarks of AD, as imaged with *in vivo* positron emission tomography, with higher education [23].

Other upstream factors including poor diet, reduced physical activity, smoking status and patterns, alcohol consumption and abuse, nutritional biomarkers including measures of anemia and vitamin D deficiency, social support and cardio-metabolic risk including elevated mid-life body mass index, blood pressure and blood glucose (or HbA1c), as well as elevated total cholesterol and measures of inflammation, have been confirmed in recent meta-analyses to be important predictors of cognitive performance, decline and incidence of dementia [16, 42–47]. Moreover, poor cognitive performance at a point in time during mid-adulthood was generally predictive of later onset dementia [48]. We found minority race status to be associated with lower SES which then predicted improved lifestyle factors in general, the latter predicting better general and cardio-metabolic health, and poorer health was associated with greater dementia risk. This pattern of associations was particularly supported in models with LE8 sub-scales with two dominant pathways (‘RACE(−) → SES(−) → DEMENTIA’ and ‘RACE(−) → SES(+) → LE8_LIFESYLE_(−) → DEMENTIA’) explaining the net excess dementia risk among Non-White adults vs. White adults. Despite poor cognitive performance predicting future dementia risk, there may be indication of reverse causality between cognitive performance and LE8_LIFESTYLE_ in particular, whereby perceived poor cognition is leading individuals to improve their diet, physical activity, and smoking habits among others. This potential reverse causation is also observed in models with individual lifestyle factors, rendering models without cognitive performance as a mediator more interpretable.

Our study has several strengths. First, our analyses were well-powered to evaluate and detect mediating effects across different racial/ethnic subgroups, overall and among males and females separately. Second, we were able to use the exact diagnosis dates for respondents due to the record linkage processes maintained by the UK Biobank investigators. Third, whereas prior work utilizing electronic health record data tends to rely on a limited set of demographic measures collected during patient encounters [49]. We were able to incorporate a broad range of characteristics across multiple domains in conjunction with electronic health record linkage, minimizing potential bias due to unmeasured confounding. Potential study limitations included residual confounding, measurement error, and potential selection bias due to missing data on cognitive performance. Furthermore, there were some limitations related to studying each racial/ethnic minority group separately, particularly African Caribbean and South Asian as contrasted with the larger group of European ancestry, for sub-types of dementia including AD and VaD, and examining in more detail those pathways through socio-economic status and cardiovascular health as measured by LE8. Nevertheless, as follow-up continues in the UK Biobank study, more incident cases of AD and VaD will allow for more granular analyses by race/ethnicity and sex. It is worth noting that we included several covariates to estimate each construct of interest among mediators and adjusted our models for potential confounding exogenous variables. Our findings are further supported by a parallel study conducted among older adults in the US [24] which revealed pathways similar to those uncovered in the current study. For example, in both studies, SES and several lifestyle factors—including diet and physical activity—were identified in explaining racial/ethnic disparities in dementia incidence. Moreover, other recent work further corroborates some of the other pathways observed in the current study, including mechanisms related to diet and social support across different income groups [33]. It is worth noting that given the contemporaneous measurement of cognitive performance and lifestyle factors among others, reverse causality whereby behavior change is driven by perceived poor cognition is observed in some of the models that included cognitive performance as a potential mediator.

Our study provides evidence for modifiable risk factors that can delay dementia onset and explain a significant portion of the SES-dementia as well as the race-dementia relationships. Our findings underscore the importance of lifestyle factors such as diet, smoking, physical activity, sleep and social support for future interventions aimed at reducing racial and socio-economic disparities in dementia.

## Supplementary Materials

Supplementary Methods and Results

Supplementary Figures

Supplementary Tables
