# Supplementary material for: Pathways explaining racial/ethnic and socio-economic disparities in dementia incidence: the UK Biobank study
Source: Aging (Albany NY). 2023 Sep 25;15(18):9310–40. doi: 10.18632/aging.205058 (PMC10564412; doi:10.18632/aging.205058)
Supplement: Supplementary Methods and Results [file aging-15-205058-s001.pdf]

SUPPLEMENTARY METHODS AND RESULTS

Supplementary Method 1: Dietary intake and other lifestyle factors

The touchscreen questionnaire of the UKB main study included twenty-nine questions regarding diet and eighteen questions related to alcohol. The touchscreen questionnaire inquired about food consumption frequency and nature, over the past year of the following food groups: cooked vegetables, salad/raw vegetables, fresh fruit, dried fruit, oily fish, other fish, processed meats, poultry, beef, lamb, pork, cheese, salt added to food, tea, water, as well as questions on the type of milk most commonly consumed, type of spread most commonly consumed, number of slices and type of bread most commonly consumed, number of bowls and type of breakfast cereal most commonly consumed, cups of coffee and type most commonly consumed, as well as questions on the avoidance of specific foods and food groups (eggs, dairy products, wheat, sugar), age last ate meat (for participants who reported never consuming processed meats, poultry, beef, lamb or pork), temperature preference of hot drinks, changes in diet in the past 5 years, and variation in diet. Four of the dietary questions originally utilized in the pilot trial were slightly altered for the main assessment phase: these were the items related to avoiding specific foods and food groups; spread type; bread type; and variation in diet.

The Healthy Diet Index (HDI) score combined several food groups in terms of quantity and frequency of consumption per week, when available to reflect the guidelines listed in Supplementary Table 2. However, those criteria were modified to fit the availability of data in the UK Biobank. Supplementary Table 3 represents the food groups that were selected, their respective coding scheme and the scoring system to reflect better diet quality, approximating the criteria in Supplementary Table 2. The touchscreen questionnaire was later validated against the 24-hr recall that was administered over time to UK Biobank participants and has shown adequate agreement in terms of ranking for each food group of interest [1].

Smoking

We utilized several fields of data to generate three tobacco exposure variables, based on the touchscreen questionnaire at the assessment centre visit, namely smoking status, environmental tobacco smoke and pack-years of smoking. Those three constructs were transformed into standardized z-scores which were then averaged into the latent construct SMOKING.

Alcohol

The touchscreen questionnaire also provided several questions related to alcohol consumption, which were quantity-frequency in nature. One question asked "About how often do you drink alcohol?" with 6 possible responses that were reverse coded to the following: 0 "never" 1 "special occasions only" 2 "1–3 times per month" 3 "1–3 times per week" 4 "3–4 times per week" 5 "daily or almost daily". The construct ALCOHOL was the standardized z-score for this item.

Physical activity

Physical activity (PA) was operationalized using a set of self-reported responses that can be used to assess mild (i.e., walking), moderate and vigorous activities based on the short form of the International Physical Activity Questions [2] in terms of frequency (# of days) per week and number of minutes per day. Those were then combined to generate MET.min/week for each category of physical activity intensity. Finally, the MET.min/week values were added together. Given that missing data does exist, addition was made on the imputed data, whereby MET.min/week per intensity were imputed where missing using chained equations. This single measured variable reflecting total MET.min/week was transformed into a standardized z-score, labelled PA and used in our pathway analyses.

Diet quality

We utilized the dietary questionnaire data category, based on a set of questions administered at the assessment visit. A measure of diet quality was constructed to approximate dietary recommendations listed in Supplementary Table 2. The criteria applied to each food or nutrient item derived the food frequency questionnaire (FFQ) to obtain an overall measure of diet quality is described in Supplementary Table 3. The resulting z-score was used to obtain the DIET construct.

Nutritional biomarkers

Vitamin D was additionally selected from the list as a nutritional biomarker that was previously shown to be inversely associated with cognitive aging [3–6]. Of the long list of hematological factors, we selected red cell distribution width (RDW) as an additional nutritional biomarker, reflecting iron metabolism, as it was previously shown to be directly associated with cognitive aging [7–9]. Thus, the z-score of RDW was multiplied by -1. The average of the two z-scores was used reflect nutritional biomarkers, or NUTR.

Social support

Three social support variables were used to operationalize SS standardized z-score. The first variable pertained to the question: "How often do you visit friends or family or have them visit you?", with potential responses reverse coded to range from 1 = "No friends/family" to 7 = "Almost daily". Intermediate responses were "Never or almost never", "Once every few months", "About once a month", "About once a week" and "2–4 times a week". Similarly, another question asked: "How often are you able to confide in someone close to you?" With no reverse coding necessary, the responses ranged from 0 = "Never or almost never" to 5 = "almost daily" and intermediate responses being "Once every few months", "About once a month", "About once a week" and "2–4 times a week". Finally, a third question asked "Which of the following do you attend once a week or more often?" and was used to count leisure and social activities among "sports and club or gym", "pub or social club", "religious group", "adult education class" and "other group activity". These three measures were then transformed into a standardized z-score and averaged into the SS measure.

Supplementary Method 2: Life's Essential 8

Life's Essential 8 was computed using guidelines from Supplementary Table 4 and all available data fields that correspond to these guidelines, while ensuring maximal sample preservation. The HDI was used for the dietary quality component, while other criteria were used that fit the guidelines well. In order to further preserve the sample and increase statistical power, two methods were available. The first one was multiple imputations using chained equations. Given the large sample to be used, this method was deemed infeasible as a main tool for the analysis. Another method that is widely used in the social science is proration [10, 11], with general guidelines for large sample to allow for up to 50% of the items to be missing per observation, as shown in Supplementary Table 1. Beyond this threshold, the entire observation was dropped from analysis. For scales that relied on totals (e.g., LE8), the row means were multiplied by the total number of items (4 for the LE8 sub-scales and 8 for the total score). This method was also applied to SES, DIET, SMOKING, ALCOHOL, NUTR, SS and HEALTH. COGN score was obtained using principal components analysis with complete cases and thus proration was not needed. In the final sample, 99.9% of participants had 2 items or less missing on the LE8 total score.

Supplementary Method 3: Health-related factors

Blood biochemistry was conducted at baseline assessment the full list of markers, included markers for liver and kidney function, systemic inflammation, lipid metabolism, glucose homeostasis and calcium metabolism among others. Some of these markers were included into the measure of allostatic load, including albumin, C-reactive protein, total cholesterol, HDL-cholesterol, and glycated hemoglobin (HbA1c). Clinical criteria summarized in Supplementary Table 5 were used to obtain risk indicators. Glycosylated hemoglobin was measured in mmol/mol and converted to %, with a cutoff of 6.4% corresponding to 41.8 mmol/mol, using high performance liquid chromatography, Bio-Rad Variant II. Nurses and phlebotomists collected blood and urine samples from participants at the assessment center after an overnight fast, which was determined largely compliant based on the pilot testing phase [14]. Among blood measures, we used total cholesterol (mg/dl), HDL-cholesterol (mg/dl), CRP (mg/dl), albumin (g/dl) and glycosylated hemoglobin (%) which were analyzed by contract laboratories [14]. Specifically, blood lipids were measured using direct enzymatic methods (Konelab, Thermo Fisher Scientific, Waltham, MA, USA). Using standard protocols, waist-to-hip ratio, radial pulse (beats/min), and systolic and diastolic blood pressure (mmHg) were measured by trained examiners. Specifically, both blood pressure and pulse rate were measured using the Omron HEM-7015IT digital blood pressure monitor [14].

BMI

The body mass index was computed at baseline assessment measured weight in kilograms divided by measured height-squared in squared-meters.

Allostatic load (AL)

Using a method described previously, [15] AL total score is an index that adds up with equal weighting (range: 0–9), cardiovascular (systolic and diastolic blood pressure, pulse rate), metabolic (total cholesterol, HDL-cholesterol, glycosylated Hb, sex-specific waist-to-hip ratio) and inflammatory (albumin and C-reactive protein (CRP)) risk indicators.

Co-morbidity index

Two data fields (134 and 135) were used to construct a variable for cancer and non-cancer co-morbidity index at the baseline assessment. These are based on self-reported data on pre-existing co-morbidities.

Self-rated health

Self-rated health (or overall health rating) was obtained as part of the touchscreen questionnaire at baseline assessment the UK Biobank. Possible responses were: 1. Excellent, 2. Good, 3. Fair, 4. Poor. The coding was left as is to reflect poorer health with higher score.

Supplementary Method 4: Cognitive test performance: assessment and scoring

The UK Biobank performed touchscreen computer assessment of cognitive performance on all participants in the case of the pairs memory test and the reaction time test. A sub-sample also completed the numeric memory test, a prospective memory task and a numeric and verbal reasoning test [24, 25]. Those tests were shown to correlate with general cognitive ability (R^2: 0.3–0.6), though generally had a lower test-retest reliability compared to reference cognitive tasks (R^2 varied from 0.4 to 0.6) [24, 25]. For our purpose, we used a total of three cognitive test scores from the pairs memory test (two scores) the reaction time test (one score), to preserve the final sample size.

Visual memory

The visual memory task involved memorizing positions of pairs of cards, followed by successfully matching them after the cards have been turned face down on the screen. In the first round, participants had 3 pairs to remember, while in the second round, they were asked to remember 6 pairs. The number of incorrect matches were of interest and Cronbach α reliability = 0.62 [26]. We have focused on the 6 pair version due to its greater difficulty. In addition, the time to complete the visual memory test was also of interest in this study.

Reaction time

Participants completed a touch screen version of the game snap and the time to match each symbol was recorded. They completed twelve rounds with the reaction time averaged across rounds. Cronbach α reliability = 0.85 [26].

Supplementary Results 1

The estimated incidence rate of all-cause dementia among men was 164 per 100,000 person-years (P-Y); among women it was 117 per 100,000 per year. For AD, incidence estimates were 63 per 100,000 P-Y among men and 54 per 100,000 P-Y among women. Dementia incidence rates for both sexes were greater among Black adults compared to White adults, which was the reverse for rates among SA and other ethnic groups. Racial/ethnic composition differed significantly across sexes, with greater percentage of Black adults among women compared to men (1.0% (F) vs. 0.8% (M)), coupled with a greater percentage SA among men vs. women (1.4% (M) vs. 1.0% (F)). Moreover, minority groups overall were younger than White adults in this sample (58.6 (NW) vs. 60.5 y (W), mean age). Household size was larger in the minority group compared to White adults (2.7 (NW) vs. 2.2 (W)) in both sexes. Importantly, non-White adults had lower SES compared to White adults (z-score: −0.28 (NW), −0.02 (W)). There were both sex and racial differences in the smoking construct. The SMOKING z-score was lower among minority groups compared to White adults (−0.025 (NW) vs. −0.004 (W)), and higher among men (−0.002) compared to women (−0.008). In contrast, men tended to consume alcohol more frequently than women, and non-White adults were less heavy consumers compared to their White counterparts. Physical activity measured in Met.min.wk^{-1} was lower among non-White adults vs. White adults, and among women compared to men. There were notable racial and ethnic differences in the NUTR z-score, owing mainly to reduced vitamin D level among non-White compared to White adults. Minority groups had poorer general and cardiometabolic health compared to White adults as did men compared with women. Minority groups combined and women performed worse on a set of cognitive test scores compared to their White and male counterparts. LE8 total, lifestyle and biological scores were markedly higher among White adults compared to non-White adults, and were also higher among women than men, suggesting a more optimal cardiovascular health among White adults and women.

Supplementary Figure 2 illustrates the results of Supplementary Tables 7 and 8, which examined similar GSEM models by substituting $LE8_{LIFESTYLE}$ with other alternative LIFESTYLE factors (DIET, PA, SMOKING, ALCOHOL, NUTR and SS), and $LE8_{BIOLOGICAL}$ with the HEALTH score. The results were comparable to the LE8 findings. Focusing on Model B, NUTR and SS were among the key antecedent mediators to HEALTH explaining racial/ethnic and SES disparities in all-cause dementia risk, both of which by being associated with reduced risk. More specifically, 'RACE_ETHN(−) → SES(+) → NUTR(−) → DEMENTIA' and 'RACE_ETHN(−) → SES(+) → SS(−) → DEMENTIA' are pathways that explained 0.9% and 0.3% of the total effect RACE_ETHN → DEMENTIA, respectively. This is in contrast with 'RACE_ETHN(−) → NUTR(−) → DEMENTIA' and 'RACE_ETHN(−) → SS(−) → DEMENTIA', which explained about 25% and 17% of the total effect, respectively. Nevertheless, the residual pathway 'RACE_ETHN → SES → DEMENTIA' in these models explained around half of the RACE_ETHN → DEMENTIA total effect. Other notable pathways by which RACE_ETHN could adversely impact dementia risk included 'RACE_ETHN(−) → SES(+)→ DIET(−) → HEALTH(+) → DEMENTIA'; 'RACE_ETHN(−) → PA(−) → HEALTH(+) → DEMENTIA'; 'RACE_ETHN(−) → SES(−) →SMOKING(+) → HEALTH(+) → DEMENTIA'; and 'RACE_ETHN(−) → SES(−) → HEALTH(+) → DEMENTIA'.

SUPPLEMENTARY REFERENCES

1. Bradbury KE, Young HJ, Guo W, Key TJ. Dietary assessment in UK Biobank: an evaluation of the performance of the touchscreen dietary questionnaire. J Nutr Sci. 2018; 7:e6. https://doi.org/10.1017/jns.2017.66 PMID:29430297

2. Committee IR. Guidelines for data processing and analysis of the International Physical Activity Questionnaire (IPAQ)—short and long forms. The International Physical Activity Questionnaire. 2005.

3. Beydoun MA, Ding EL, Beydoun HA, Tanaka T, Ferrucci L, Zonderman AB. Vitamin D receptor and megalin gene polymorphisms and their associations with longitudinal cognitive change in US adults. Am J Clin Nutr. 2012; 95:163–78. https://doi.org/10.3945/ajcn.111.017137 PMID:22170372

4. Beydoun MA, Hossain S, Fanelli-Kuczmarski MT, Beydoun HA, Canas JA, Evans MK, Zonderman AB. Vitamin D Status and Intakes and Their Association With Cognitive Trajectory in a Longitudinal Study of Urban Adults. J Clin Endocrinol Metab. 2018; 103:1654–68. https://doi.org/10.1210/jc.2017-02462 PMID:29409006

5. Beydoun MA, Shaked D, Hossain S, Beydoun HA, Katzel LI, Davatzikos C, Gullapalli RP, Seliger SL, Erus G, Evans MK, Zonderman AB, Waldstein SR. Vitamin D, Folate, and Cobalamin Serum Concentrations Are Related to Brain Volume and White Matter Integrity in Urban Adults. Front Aging Neurosci. 2020; 12:140. https://doi.org/10.3389/fnagi.2020.00140 PMID:32523528

6. Beydoun MA, Tajuddin SM, Dore GA, Canas JA, Beydoun HA, Evans MK, Zonderman AB. Vitamin D Receptor and Megalin Gene Polymorphisms Are Associated with Longitudinal Cognitive Change among African-American Urban Adults. J Nutr. 2017; 147:1048–62. https://doi.org/10.3945/jn.116.244962 PMID:28446629

7. Beydoun MA, Hossain S, Beydoun HA, Shaked D, Weiss J, Evans MK, Zonderman AB. Red Cell Distribution Width Is Directly Associated with Poor Cognitive Performance among Nonanemic, Middle-Aged, Urban Adults. J Nutr. 2020; 150:128–39. https://doi.org/10.1093/jn/nxz182 PMID:31912144

8. Beydoun MA, Hossain S, MacIver PH, Srinivasan D, Beydoun HA, Maldonado AI, Katzel LI, Davatzikos C, Gullapalli RP, Seliger SL, Erus G, Evans MK, Zonderman AB, Waldstein SR. Red Cell Distribution Width, Anemia, and Brain Volumetric Outcomes Among Middle-Aged Adults. J Alzheimers Dis. 2021; 81:711–27. https://doi.org/10.3233/JAD-201386 PMID:33814435

9. Beydoun MA, Shaked D, Hossain S, Weiss J, Beydoun HA, Maldonado AI, Katzel LI, Davatzikos C, Gullapalli RP, Seliger SL, Erus G, Evans MK, Zonderman AB, Waldstein SR. Red cell distribution width, anemia and their associations with white matter integrity among middle-aged urban adults. Neurobiol Aging. 2021; 105:229–40. https://doi.org/10.1016/j.neurobiolaging.2021.05.004 PMID:34120091

10. Mazza GL, Enders CK, Ruehlman LS. Addressing Item-Level Missing Data: A Comparison of Proration and Full Information Maximum Likelihood Estimation. Multivariate Behav Res. 2015; 50:504–19. https://doi.org/10.1080/00273171.2015.1068157 PMID:26610249

11. Wu W, Gu F, Fukui S. Combining proration and full information maximum likelihood in handling missing data in Likert scale items: A hybrid approach. Behav Res Methods. 2022; 54:922–40. https://doi.org/10.3758/s13428-021-01671-w PMID:34357540

12. Hayman LL, Martyn-Nemeth P. A New Metric for Promoting Cardiovascular Health: Life's Essential 8. J Cardiovasc Nurs. 2022; 37:509–11. https://doi.org/10.1097/JCN.0000000000000946 PMID:36037296

13. Lloyd-Jones DM, Allen NB, Anderson CAM, Black T, Brewer LC, Foraker RE, Grandner MA, Lavretsky H, Perak AM, Sharma G, Rosamond W, American Heart Association. Life's Essential 8: Updating and Enhancing the American Heart Association's Construct of Cardiovascular Health: A Presidential Advisory From the American Heart Association. Circulation. 2022; 146:e18–43. https://doi.org/10.1161/CIR.0000000000001078 PMID:35766027

14. UK Biobank. (2007). UK Biobank: Protocol for a large-scale prospective epidemiological resource.

15. Seeman T, Merkin SS, Crimmins E, Koretz B, Charette S, Karlamangla A. Education, income and ethnic differences in cumulative biological risk profiles in a national sample of US adults: NHANES III (1988-1994). Soc Sci Med. 2008; 66:72–87. https://doi.org/10.1016/j.socscimed.2007.08.027 PMID:17920177

16. Visser M, Kritchevsky SB, Newman AB, Goodpaster BH, Tylavsky FA, Nevitt MC, Harris TB. Lower serum albumin concentration and change in muscle mass: the Health, Aging and Body Composition Study. Am J Clin Nutr. 2005; 82:531–7. https://doi.org/10.1093/ajcn.82.3.531 PMID:16155264

17. Ridker PM. Cardiology Patient Page. C-reactive protein: a simple test to help predict risk of heart attack and stroke. Circulation. 2003; 108:e81–5. https://doi.org/10.1161/01.CIR.0000093381.57779.67 PMID:14504253

18. Alberti KG, Zimmet PZ. Definition, diagnosis and classification of diabetes mellitus and its complications. Part 1: diagnosis and classification of diabetes mellitus provisional report of a WHO consultation. Diabet Med. 1998; 15:539–53. https://doi.org/10.1002/(SICI)1096-9136(199807)15:7%3c539::AID-DIA668%3e3.0.CO;2-S PMID:9686693

19. Expert Panel on Detection, Evaluation, and Treatment of High Blood Cholesterol in Adults. Executive Summary of The Third Report of The National Cholesterol Education Program (NCEP) Expert Panel on Detection, Evaluation, And Treatment of High Blood Cholesterol In Adults (Adult Treatment Panel III). JAMA. 2001; 285:2486–97. https://doi.org/10.1001/jama.285.19.2486 PMID:11368702

20. Golden S, Boulware LE, Berkenblit G, Brancati F, Chander G, Marinopoulos S, Paasche-Orlow M, Powe N, Rami T. Use of glycated hemoglobin and microalbuminuria in the monitoring of diabetes mellitus. Evid Rep Technol Assess (Summ). 2003; :1–6. PMID:15040142

21. Osei K, Rhinesmith S, Gaillard T, Schuster D. Is glycosylated hemoglobin A1c a surrogate for metabolic syndrome in nondiabetic, first-degree relatives of African-American patients with type 2 diabetes? J Clin Endocrinol Metab. 2003; 88:4596–601. https://doi.org/10.1210/jc.2003-030686 PMID:14557428

22. Seccareccia F, Pannozzo F, Dima F, Minoprio A, Menditto A, Lo Noce C, Giampaoli S, Malattie Cardiovascolari Aterosclerotiche Istituto Superiore di Sanita Project. Heart rate as a predictor of mortality: the MATISS project. Am J Public Health. 2001; 91:1258–63. https://doi.org/10.2105/ajph.91.8.1258 PMID:11499115

23. Lenfant C, Chobanian AV, Jones DW, Roccella EJ, Joint National Committee on the Prevention, Detection, Evaluation, and Treatment of High Blood Pressure. Seventh report of the Joint National Committee on the Prevention, Detection, Evaluation, and Treatment of High Blood Pressure (JNC 7): resetting the hypertension sails. Hypertension. 2003; 41:1178–9. https://doi.org/10.1161/01.HYP.0000075790.33892.AE PMID:12756222

24. Sinclair LI, Ball HA, Bauermeister S, Gallacher JEJ, Bolea-Alamanac BM. Recurrent depression has persistent effects on cognition but this does not appear to be mediated by neuroinflammation. J Affect Disord. 2022; 306:232–9. https://doi.org/10.1016/j.jad.2022.03.043 PMID:35337923

25. Fawns-Ritchie C, Deary IJ. Reliability and validity of the UK Biobank cognitive tests. PLoS One. 2020; 15:e0231627. https://doi.org/10.1371/journal.pone.0231627 PMID:32310977

26. Hagenaars SP, Harris SE, Davies G, Hill WD, Liewald DC, Ritchie SJ, Marioni RE, Fawns-Ritchie C, Cullen B, Malik R, METASTROKE Consortium, International Consortium for Blood Pressure GWAS, SpiroMeta Consortium, CHARGE Consortium Pulmonary Group, CHARGE Consortium Aging and Longevity Group, et al. Shared genetic aetiology between cognitive functions and physical and mental health in UK Biobank (N=112 151) and 24 GWAS consortia. Mol Psychiatry. 2016; 21:1624–32. https://doi.org/10.1038/mp.2015.225 PMID:26809841
