# Supplementary material for: Pathways explaining racial/ethnic and socio-economic disparities in dementia incidence: the UK Biobank study
Source: Aging (Albany NY). 2023 Sep 25;15(18):9310–40. doi: 10.18632/aging.205058 (PMC10564412; doi:10.18632/aging.205058)
Supplement: Supplementary Figures [file aging-15-205058-s002.pdf]

SUPPLEMENTARY FIGURES

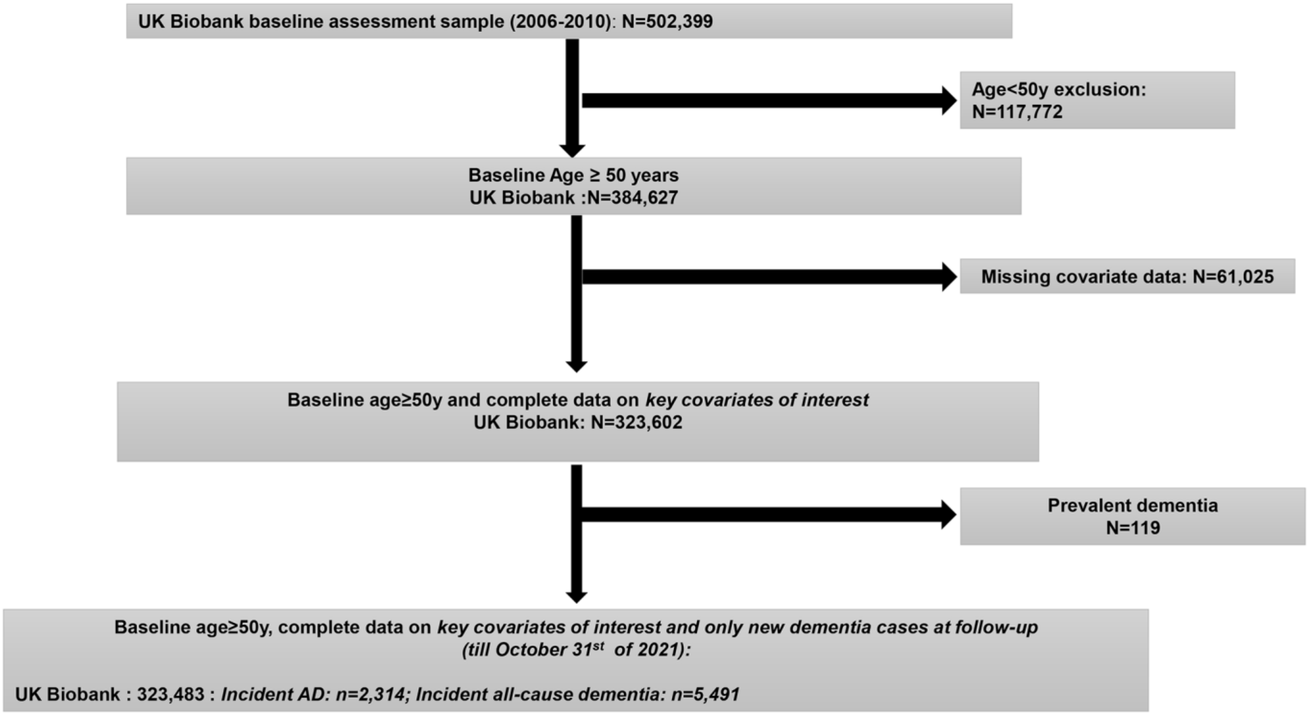

Supplementary Figure 1. Participant flowchart: The UK biobank 2006–2021. Abbreviations: AD: Alzheimer's disease; N: Sample size; UK: United Kingdom.

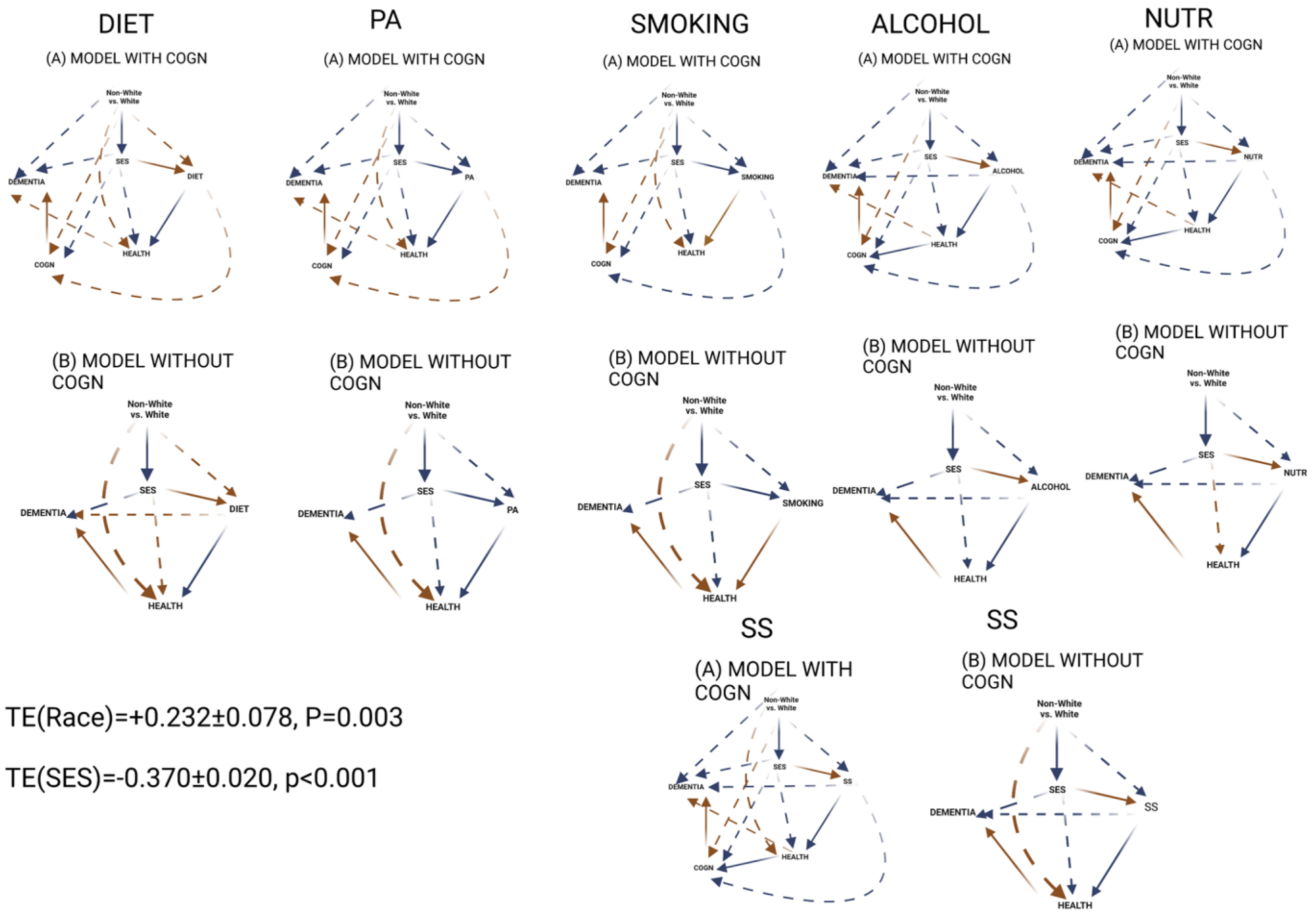

Supplementary Figure 2. GSEM findings from models with alternative mediators, HEALTH and COGN. Abbreviations*:* AD: Alzheimer's Disease; ALCOHOL: Alcohol consumption z-score; COGN: Poor cognitive performance z-score; DIET: diet quality z-score; HEALTH: Poor cardio-metabolic and general health z-score; PA: Physical Activity z-score; NUTR: Nutritional biomarker z-score; SES: Socio-economic status z-score; SMOKING: Smoking z-score; SS: Social Support z-score; TE: Total Effect. Red lines: Positive associations; Blue lines: Inverse associations; Solid lines: Within hypothesized pathway; Dashed lines: Outside hypothesized pathway.
