# Supplementary material for: Pathways explaining racial/ethnic and socio-economic disparities in dementia incidence: the UK Biobank study
Source: Aging (Albany NY). 2023 Sep 25;15(18):9310–40. doi: 10.18632/aging.205058 (PMC10564412; doi:10.18632/aging.205058)
Supplement: Supplementary Tables [file aging-15-205058-s003.pdf]

SUPPLEMENTARY TABLES

Supplementary Table 1. Proration of mediators including LE8 sub-scales.

Items	Number of missing items/participant allowed for prorating
1	0
2	1
3	1
4	2
5	2
6	3
7	3
8	4
9	4
10	5
11	5
12	6

Supplementary Table 2. Goals and guidelines used to construct the healthy diet score.

Consume more	Goal*	One serving equals…
**Fruits**	3 servings/d	1 medium-sized fruit; ½ cup of fresh, frozen, or unsweetened canned fruit; ½ cup of dried fruit; ½ cup of 100% juice
**Nuts, seeds**	4 servings/wk	1 ounce
**Vegetables, including legumes (excluding russet or white potatoes)**	3 servings/d	1 cup of raw leafy vegetables; ½ cup of cut-up raw vegetables, cooked vegetables, or 100% vegetable juice
**Whole grains†**	3 servings/d, in place of refined grains	1 slice of whole-grain bread; 1 cup of high-fiber, whole-grain cereal; ½ cup of cooked whole-grain rice, pasta, or cereal
**Fish, shellfish**	≥2 servings/wk	3.5 ounces (100 g)
**Dairy products, especially yogurt and cheese‡**	2–3 servings/d	1 cup of milk or yogurt; 1 ounce of cheese
**Vegetable oils**	2–6 servings/d	1 teaspoon oil, 1 tablespoon vegetable spread
**Consume less**		
**Refined grains, starches, added sugars†**	No more than 1–2 servings/d	
**Processed meats**	No more than 1 serving/wk	1.75 ounces (50 g)
**Unprocessed red meats**	No more than 1–2 servings/wk	3.5 ounces (100 g)
**Industrial trans fat§**	Don't eat	Any food containing or made with partially hydrogenated vegetable oil
**Sugar-sweetened beverages**	Don't drink	8 ounces of beverage; 1 small sweet, pastry, or dessert
**Sodium**	No more than 2000 mg/d	n/a

Source: https://www.ahajournals.org/doi/10.1161/CIRCULATIONAHA.115.018585#d3e341. *Based on a 2000 kcal/d diet. Servings should be adjusted accordingly for higher or lower energy consumption. †As a practical rule-of-thumb for selecting healthful whole grains and avoiding carbohydrate-rich products high in starches and added sugars, the ratio of total carbohydrate to dietary fiber (g/serving of each) appears useful. Foods with ratios <10:1 are preferable; i.e., food containing

at least 1 g of fiber for every 10 g of total carbohydrate. In addition, minimally processed whole grains (e.g., steel-cut oats, stone-ground bread) are generally preferable to finely milled whole grains (e.g., many commercial whole-grain breads and breakfast cereals) because of the larger glycemic responses of the latter. [‡]Current evidence does not permit clear differentiation of whether low-fat or whole-fat products are superior for cardiometabolic health. Other characteristics, such as probiotic content or fermentation, may be far more relevant than fat content. [§]The US Food and Drug Administration recently ruled that the use of partially hydrogenated vegetable oils is no longer "generally regarded as safe," which should effectively eliminate the majority of industrial trans fats from the US food supply. Several countries including Denmark, Argentina, Austria, Iceland, and Switzerland have effectively eliminated the use of partially hydrogenated vegetable oils through direct legislation on the amounts of allowable trans fats in foods. Small amounts of certain trans fatty acids may be formed through other industrial processes, including oil deodorization and high-temperature cooking; the health effects of these trace industrial trans fats require careful investigation.

Supplementary Table 3. Healthy Diet Index, HDI, using touchscreen questionnaire in the UK Biobank study[†,§].

Food group/ nutrient item	UKB fields used	Definition of meeting criterion	Criteria and scoring
**Consume more**			
Fruits, fresh or dried	1309 and 1319	≥3 servings per day including fresh and dried fruits<br>1 piece of dried fruit (e.g., apricot) ~=2.5 TBSP, 1 TBSP: 0.063 cups; ½ cup of dried fruit (1 serving) is 3 pieces of dried fruit.<br>1 medium sized fruit is one serving.	1: meets criterion,<br>0: does not meet criterion
Vegetables, salad/cooked	1289 and 1299	≥3 servings per day<br>Including salad, raw and cooked<br>1 cup of raw leafy vegetables is 16 TBSP. ½ cup of cooked or non-leafy raw vegetables is 8 TBSP.<br>1 serving of raw leafy or non-leafy vegetables is on average ~=12 TBSP; 1 serving of cooked vegetables is ~=8 TBSP	1: meets criterion,<br>0: does not meet criterion
Whole grains		≥3 servings per day	1: meets criterion,<br>0: does not meet criterion
**Slices of bread**	1438 and 1448	Daily slices of wholemeal or wholegrain bread (servings per day), convert from weekly slices.	
**Cereal**	1458 and 1448	Daily bowls of whole wheat cereal as servings/day (bran cereal, biscuit cereal, oat cereal and muesli), convert from weekly bowls.	
Fish shellfish	1329 and 1339	Sum weekly frequencies to obtain total servings/week.<br>≥2 servings/wk	1: meets criterion,<br>0: does not meet criterion
**Oily fish**	…	…	
**Non-oily fish**	…	…	
Dairy products	6114, 1408 and 1418	Reporting consumption of two milk items and eating cheese once a day to meet the 2–3 servings/day criterion.	1: meets criterion,<br>0: does not meet criterion
**Milk**	…		
**Cheese**	…		
Vegetable oil	2654	Reporting use of olive oil or polyunsaturated/sunflower oil (yes: 1, 0: no)	1: meets criterion,<br>0: does not meet criterion
**Consume less**			
Refined grains, starches, added sugars[†]	1438 and 1448	Follow a similar coding scheme as for whole grains but select non-whole grains;<br><1.5 servings per day	1: meets criterion,<br>0: does not meet criterion
Processed meats	1349	Once a week or less would meet the criterion.	1: meets criterion,

			0: does not meet criterion
Unprocessed red meats	1369, 1379, and 1389	Summation of frequency of consumption across three types of red meats (lamb/mutton, beef or pork).<br><3 on the summation corresponds to the criterion of <1–2 servings per week.	1: meets criterion,<br>0: does not meet criterion
Industrial trans fat[§]	1428	Never use spread, e.g., butter or margarine etc. would meet the criterion	1: meets criterion,<br>0: does not meet criterion
Sugar-sweetened beverages	6144	Never eat sugar or food/drink containing sugar would meet the criterion	1: meets criterion,<br>0: does not meet criterion
Sodium	1478	Salt added to food, never or rarely would meet the criterion	1: meets criterion,<br>0: does not meet criterion

Source: https://biobank.ndph.ox.ac.uk/showcase/label.cgi?id=100052. Stata code can be made available upon request.

Supplementary Table 4. Definition and scoring approach for quantifying cardiovascular health, as per the American Heart Association's Life's Essential 8 score [12, 13], and as applied in the National Health and Nutrition Examination Surveys, 2013–2018.

**Domain**	**CVH metric**	**Method of measurement**	**Quantification of CVH metric - adults (≥20 years)**
**Health Behaviors**	**Diet**	**Measurement: Self-reported daily intake of a DASH-style eating pattern**<br>**Example tools for measurement: DASH diet score (populations)**	Quantiles of DASH-style diet adherence<br>**Scoring (Population):**<br><u>Points</u> <u>Quantile</u><br>100 ≥95th %ile (top/ideal diet)<br>80 75th – 94th %ile<br>50 50th – 74th %ile<br>25 25th – 49th %ile<br>0 1st – 24th %ile (bottom/least ideal quartile)
	**Physical activity**	**Measurement: Self-reported minutes of moderate or vigorous physical activity per week**<br>**Example tools for measurement:** NHANES PAQ-K questionnaire	**Metric: Minutes of moderate (or greater) intensity activity per week**<br>**Scoring:**<br><u>Points</u> <u>Minutes</u><br>100≥150<br>90 120 – 149<br>80 90 – 119<br>60 60 – 89<br>40 30 – 59<br>20 1 – 29<br>0 0
	**Nicotine exposure**	**Measurement:** Self-reported use of cigarettes or inhaled nicotine- delivery system<br>**Example tools for measurement:** NHANES SMQ	**Metric: Combustible tobacco use and/or inhaled NDS use; or secondhand smoke exposure**<br>**Scoring:**<br><u>Points</u> <u>Status</u><br>100 Never smoker 75Former smoker, quit≥5 yrs<br>50 Former smoker, quit 1 - <5 yrs<br>25 Former smoker, quit <1 year, or currently using inhaled NDS<br>0 Current smoker<br>Subtract 20 points (unless score is 0) for living with active indoor smoker in home
	**Sleep health**	**Measurement:** Self-reported average hours of sleep per night<br>**Example tools for measurement:** "On average, how many hours of sleep do you get per night?" Consider objective sleep/actigraphy data from wearable technology, if available	**Metric:** Average hours of sleep per night<br>**Scoring:**<br><u>Points</u> <u>Level</u><br>100 7 – <9<br>90 9 – <10<br>70 6 – <7<br>40 5 – <6 or ≥10<br>20 4 – <5<br>0 <4

**Health Factors**	**Body mass index**	**Measurement:** Body weight (kg) divided by height squared (m2)<br>**Example tools for measurement:** Objective measurement of height and weight	**Metric: Body mass index (kg/m^2)**<br>**Scoring:**<br>Points Level<br>100 <25<br>70 25.0 – 29.9<br>30 30.0 – 34.9<br>15 35.0 – 39.9<br>0 ≥ 40.0
	**Blood lipids**	**Measurement:** Plasma total and HDL-cholesterol with calculation of non-HDL-cholesterol<br>**Example tools for measurement:** Fasting or non-fasting blood sample	**Metric: Non-HDL-cholesterol (mg/dL)**<br>**Scoring:**<br>Points Level<br>100 <130<br>60 130 – 159<br>40 160 – 189<br>20 190 – 219<br>0 ≥220<br>If drug-treated level, subtract 20 points
	**Blood glucose**	**Measurement: Fasting blood glucose or casual hemoglobin A1c Example tools for measurement: Fasting (FBG, HbA1c) or non- fasting (HbA1c) blood sample**	**Metric: Fasting blood glucose (mg/dL) or Hemoglobin A1c (%)**<br>**Scoring:**<br>Points Level<br>100 No history of diabetes and FBG <100 (or HbA1c < 5.7)<br>60 No diabetes and FBG 100 – 125 (or HbA1c 5.7–6.4) (Pre-diabetes)<br>40 Diabetes with HbA1c <7.0<br>30 Diabetes with HbA1c 7.0 – 7.9<br>20 Diabetes with HbA1c 8.0 – 8.9<br>10 Diabetes with Hb A1c 9.0 – 9.9<br>0 Diabetes with HbA1c ≥10.0
	**Blood pressure**	**Measurement:** Appropriately measured systolic and diastolic blood pressure<br>**Example tools for measurement:** Appropriately sized blood pressure cuff	**Metric:** Systolic and diastolic blood pressure (mm Hg)<br>**Scoring:**<br>Points Level<br>100 <120/<80 (Optimal)<br>75 120−129/<80 (Elevated)<br>50 130−139 or 80–89 (Stage I HTN)<br>25 140−159 or 90–99<br>0 ≥ 160 or ≥100

Supplementary Table 5. Allostatic load indicator criteria [15].

	High-risk clinical
Albumin (g/dL)	<3.8 [16]
C-reactive protein (mg/dL)	≥0.3 [17]
Waist: Hip Ratio	>0.9 for males; >0.85 for females [18]
Total cholesterol (mg/dL)	≥240 [19]
HDL-C (mg/dL)	<40 [19]
Glycated hemoglobin (%)	≥6.4 [20, 21]
Resting heart rate (beat/min)	≥90 [22]
Systolic BP	≥140 [23]
Diastolic BP	≥90 [23]

Abbreviations: BP: Blood Pressure; HDL: High Density Lipoprotein-Cholesterol.

Supplementary Table 6. Study sample characteristics by sex: The UK Biobank 2006–2021[a].

	Males, *n* = 148,958				Females, *n* = 173,525				P_{sex}
**Study sample characteristics**	**All males**	**White**	**Non-White**	***P***	**All females**	**White**	**Non-White**	***P***	
**Socio-demographic**									
**Baseline age, y**	60.7 ± 5.4	60.8 ± 5.4	59.1 ± 5.7	<0.001	60.1 ± 5.4	60.2 ± 5.4	58.1 ± 5.5	<0.001	**<0.001**
**Sex, % female**	0.0	0.0	0.0	—	100.0	100.0	100.0	—	—
**Race/ethnicity**				—				—	
White	96.4	100.0	0.0	—	96.4	100.0	0.0	—	<0.001
Black	0.8	0.0	22.6	—	1.0	0.0	27.5	—	—
South Asian	1.4	0.0_	39.8	—	1.0	0.0	28.3	—	<0.001
Other	1.4	0.0	37.6	—	1.6	0.0	44.2		0.45
**Household size**	2.3 ± 1.2	2.3 ± 1.1	2.9 ± 1.5	<0.001	2.2 ± 1.2	2.1 ± 1.1	2.6 ± 1.7	<0.001	<0.001
**Socio-economic status**									
**Education**									
Low	24.1	24.2	21.6	—	19.8	19.6	24.9	—	—
Intermediate	34.8	35.1	27.9	0.001	43.7	44.2	32.1	<0.001	<0.001
High	41.1	40.7	51.4	<0.001	36.5	36.2	43.0	<0.001	<0.001
**Income**				<0.001				<0.001	<0.001
Less than £18,000	22.6	22.4	28.1	—	27.8	27.7	32.3	—	—
£18,000–£29,999	26.9	26.9	27.3	—	29.0	29.0	28.3	—	—
£30,000–£51,999	25.8	25.9	22.8	—	24.0	24.0	22.2	—	—
£52,000–£100,000	19.5	19.6	16.7	—	15.5	15.5	13.6	—	—
greater than £100,000	5.2	5.2	5.1	—	3.7	3.7	3.6		
**TDI**	−1.54 ± 2.99	−1.62 ± 2.9	0.41 ± 3.53	<0.001	−1.57 ± 2.91	−1.65 ± 2.85	0.53 ± 3.43	<0.001	0.013
**SES z-score**	−0.01 ± 0.73	0.00 ± 0.72	−0.23 ± 0.80	<0.001	−0.05 ± 0.68	−0.03 ± 0.68	−0.33 ± 0.78	<0.001	<0.001
**Lifestyle factors**									
**Smoking**									
**Smoking status**									
Never	78.5	78.4	81.4	—	83.8	83.6	89.9	—	—
Former	10.6	10.8	5.4	<0.001	8.5	8.7	3.1	<0.001	<0.001
Current	10.9	10.8	13.2	<0.001	7.7	7.8	7.1	0.001	<0.001
Environmental tobacco smoke	0.97 ± 5.4	0.97 ± 5.4	1.02 ± 4.72	0.48	0.81 ± 5.1	0.80 ± 5.1	1.04 ± 4.73	<0.001	<0.001
Pack-years of tobacco smoke	0.10 ± 0.30	0.10 ± 0.30	0.07 ± 0.22	<0.001	0.07 ± 0.22	0.07 ± 0.22	0.04 ± 0.16	<0.001	<0.001
**SMOKING z-score**	−0.002 ± 0.481	−0.000 ± 0.482	−0.050 ± 0.454	<0.001	−0.008 ± 0.405	−0.008 ± 0.406	−0.004 ± 0.383	0.51	<0.001
**Alcohol consumption**									
**Alcohol consumption frequency**									
0 "never"	5.5	4.9	21.2	—	8.8	8.1	27.4	—	—
1 "special occasions only"	6.8	6.5	16.5	<0.001	14.8	14.2	30.0	<0.001	<0.001
2 "1—3 times per month"	8.0	8.0	10.1	<0.001	12.5	12.5	11.6	<0.001	0.020
3 "1—3 times per week"	24.5	24.6	21.4	<0.001	25.0	25.3	15.4	<0.001	<0.001
4 "3—4 times per week"	26.8	27.2	15.6	<0.001	21.6	21.5	8.9	<0.001	<0.001
5 "daily or almost daily"	28.4	28.9	15.2	<0.001	17.9	18.4	6.8	<0.001	<0.001
**ALCOHOL z-score**	+0.20 ± 0.94	0.23 ± 0.92	−0.49 ± 1.15	<0.001	−0.17 ± 1.00	−0.14 ± 1.00	−0.96 ± 1.02	<0.001	<0.001
**Physical activity, PA**									
**PA, Met.min.wk^{-1}**	2,169 ± 3.189	2,180 ± 3,194	1,853 ± 3,023	<0.001	1,787 ± 2,437	1,790 ± 2,431	1,703 ± 2,580	0.005	
**PA z-score**	0.07 ± 1.13	0.08 ± 1.13	−0.039 ± 1.07	<0.001	−0.06 ± 0.86	−0.06 ± 0.86	−0.09 ± 0.92	0.005	<0.001
**Diet quality**									
**HDI total score**	4.81 ± 1.56	4.79 ± 1.57	5.13 ± 1.49	<0.001	5.37 ± 1.39	5.36 ± 1.39	5.57 ± 1.35	<0.001	<0.001
**DIET z-score**	−0.20 ± 1.04	−0.21 ± 1.04	+0.01 ± 0.99	<0.001	+0.17 ± 0.93	0.17 ± 0.93	0.31 ± 0.90	<0.001	<0.001
**Nutritional Biomarkers**									
**25-hydroxyvitamin D**	49.7 ± 21.1	50.3 ± 20.9	33.8 ± 17.9	<0.001	49.6 ± 20.7	50.0 ± 20.6	36.9 ± 18.1	<0.001	0.016
**Red cell distribution width**	13.5 ± 0.9	13.5 ± 0.9	13.7 ± 1.1	<0.001	13.5 ± 1.0	13.5 ± 0.9	13.9 ± 1.3	<0.001	0.002
**NUTR z-score**	0.004 ± 0.733	+0.023 ± 0.723	−0.500 ± 0.823	<0.001	−0.005 ± 0.777	0.013 ± 0.766	−0.49 ± 0.91	<0.001	0.001
**Social Support**									
**"How often do you visit friends or family or have them visit you?"**	5.09 ± 1.16	5.10 ± 1.16	4.81 ± 1.20	<0.001	5.42 ± 1.09	5.44 ± 1.08	4.86 ± 1.21	<0.001	<0.001
**"How often are you able to confide in someone close to you?"**	1.00 ± 0.83	1.00 ± 0.83	0.91 ± 0.80	<0.001	1.08 ± 0.90	1.08 ± 0.90	0.98 ± 0.85	<0.001	<0.001
**"Which of the following do you attend once a week or more often?"**	3.43 ± 2.02	3.44 ± 2.02	2.93 ± 2.04	<0.001	3.65 ± 1.76	3.67 ± 1.75	3.12 ± 1.92	<0.001	<0.001
**SS z-score**	−0.089 ± 0.645	−0.082 ± 0.642	−0.293 ± 0.671	<0.001	0.075 ± 0.614	0.086 ± 0.609	−0.221 ± 0.664	<0.001	<0.001
**Cardio-metabolic and general health-related factors**									
**Body mass index, kg.m^{-1}**	27.9 ± 4.2	27.9 ± 4.2	27.5 ± 4.1	<0.001	27.2 ± 5.1	27.1 ± 5.0	28.1 ± 5.6	<0.001	<0.001
**Allostatic load**	2.42 ± 1.35	2.41 ± 1.35	2.46 ± 1.38	0.019	1.83 ± 1.35	1.82 ± 1.34	2.02 ± 1.40	<0.001	<0.001
**Co-morbidity index**	2.07 ± 1.86	2.07 ± 1.86	2.08 ± 1.84	0.91	2.15 ± 2.00	2.15 ± 2.01	2.16 ± 1.98	0.50	<0.001

**Self-rated health**				<0.001				<0.001	<0.001
**Excellent**	15.8	16.0	11.9		17.1	17.4	10.7		
**Good**	56.9	57.0	52.4		60.8	61.1	53.9		
**Fair**	22.5	22.2	28.8		18.7	18.3	28.8		
**Poor**	4.9	4.8	6.9		3.4	3.3	6.6		
**HEALTH z-score**	0.077 ± 0.660	0.076 ± 0.661	0.117 ± 0.650	<0.001	−0.066 ± 0.704	−0.072 ± 0.702	0.104 ± 0.743	<0.001	<0.001
**Cognitive performance**									
**Reaction Time**	6.31 ± 0.19	6.31 ± 0.18	6.40 ± 0.22	<0.001	6.34 ± 0.18	6.34 ± 0.18	6.42 ± 0.22	<0.001	<0.001
**Pairs matching, errors**	0.71 ± 0.71	0.70 ± 0.70	1.00 ± 0.75	<0.001	0.72 ± 0.69	0.71 ± 0.69	0.99 ± 0.71	<0.001	<0.001
**Pairs matching, time to complete**	5.34 ± 0.37	5.33 ± 0.37	5.57 ± 0.47	<0.001	5.36 ± 0.37	5.35 ± 0.36	5.58 ± 0.46	<0.001	<0.001
**COGN z-score**	−0.043 ± 0.764	−0.062 ± 0.750	+0.456 ± 0.940	<0.001	0.037 ± 0.746	0.019 ± 0.73	0.504 ± 0.896	<0.001	<0.001
**LE8**									
**Total score**	493.3 ± 93.0	493.7 ± 93.0	483.7 ± 94.0	<0.001	510.1 ± 97.1	510.8 ± 97.1	493.2 ± 96.1	<0.001	<0.001
**Biological score**	243.7 ± 62.0	243.8 ± 61.9	239.5 ± 64.3	<0.001	248.7 ± 69.0	249.3 ± 68.3	233.1 ± 73.7	<0.001	<0.001
**Lifestyle score**	249.6 ± 63.8	249.8 ± 63.7	243.9 ± 64.9	<0.001	261.2 ± 62.3	261.3 ± 62.3	258.7 ± 60.8	0.001	<0.001
**Incidence proportion**									
**All-cause dementia**	1.99 (*n* = 2,980)	1.99 (*n* = 2,882)	1.81 (*n* = 98)	0.34	1.45 (*n* = 2,511)	1.46 (*n* = 2,439)	1.14 (*n* = 72)	0.040	<0.001
**AD dementia**	0.76 (*n* = 1,147)	0.77 (*n* = 1,111)	0.66 (*n* = 36)	0.39	0.67 (*n* = 1,167)	0.68 (*n* = 1,134)	0.53 (*n* = 33)	0.14	0.002
**Incident rates, per 100,000 P-Y**									
**All-cause dementia**	164	164	214 (Black)<br>132 (SA)<br>134 (Others)		117	117	130 (Black)<br>74 (SA)<br>85 (Others)	—	—
**AD dementia**	63	63	69 (Black)<br>58 (SA)<br>45 (Others)		54	54	63 (Black)<br>41 (SA)<br>32 (Others)	—	—

Abbreviations: AD: Alzheimer's Disease; ALCOHOL: Alcohol consumption z-score; COGN: Poor cognitive performance z-score; DIET: diet quality z-score; HEALTH: Poor cardio-metabolic and general health z-score; PA: Physical Activity z-score; NUTR: Nutritional biomarker z-score; SES: Socio-economic status z-score; SMOKING: Smoking z-score; SS: Social Support z-score. [a]Values are means +/− SD or percentages.

Supplementary Table 7. Generalized Structural Equations models (GSEM) for racial/ethnic disparities in all-cause dementia: mediation through SES, alternative lifestyle factors (LIFESTYLE), health-related factors (HEALTH) and cognitive performance (COGN): The UK Biobank 2006–2021[a].

	**LIFESYLTE**					
	**DIET**	**PA**	**SMOKING**	**ALCOHOL**	**NUTR**	**SS**
Main pathway						
RACE_ETHN→SES (β_{12})	**−0.351 ± 0.006*****	**−0.351 ± 0.006*****	**−0.351 ± 0.006*****	**−0.351 ± 0.006*****	**−0.351 ± 0.006*****	**−0.351 ± 0.006*****
SES→LIFESTYLE (β_{23})	**+0.192 ± 0.003*****	**−0.059 ± 0.003*****	**−0.152 ± 0.002*****	**+0.305 ± 0.002*****	**+0.102 ± 0.002*****	**+0.086 ± 0.002*****
LIFESTYLE → HEALTH (β_{34})	**−0.081 ± 0.001*****	**−0.091 ± 0.001*****	**+0.046 ± 0.002*****	**−0.094 ± 0.001*****	**−0.168 ± 0.002*****	**−0.063 ± 0.002*****
HEALTH→COGN(β_{45})	−0.002 ± 0.002	−0.001 ± 0.002	−0.003 ± 0.002	**−0.010 ± 0.002*****	**−0.007 ± 0.002*****	**−0.006 ± 0.002*****
COGN → DEMENTIA (β_{56})	**+0.416 ± 0.017*****	**+0.416 ± 0.017*****	**+0.416 ± 0.017*****	**+0.412 ± 0.017*****	**+0.414 ± 0.017*****	**+0.410 ± 0.017*****
Selected direct effects on final outcomes						
RACE_ETHN→DEMENTIA(β_{16})	**−0.167 ± 0.080***	**−0.156 ± 0.079***	**−0.157 ± 0.079***	**−0.202 ± 0.080***	**−0.209 ± 0.080****	**−0.184 ± 0.079***
SES → DEMENTIA(β_{26})	**−0.220 ± 0.020*****	**−0.215 ± 0.020*****	**−0.216 ± 0.020*****	**−0.196 ± 0.020*****	**−0.209 ± 0.020*****	**−0.203 ± 0.020***
LIFESTYLE → DEMENTIA(β_{36})	+0.026 ± 0.014	+0.011 ± 0.013	−0.004 ± 0.019	**−0.075 ± 0.014*****	**−0.111 ± 0.017*****	**−0.163 ± 0.021*****
HEALTH → DEMENTIA(β_{46})	**+0.408 ± 0.019*****	**+0.407 ± 0.019*****	**+0.404 ± 0.019*****	**+0.388 ± 0.019*****	**+0.378 ± 0.020*****	**+0.395 ± 0.019*****
Other effects between endogenous variables						
SES→HEALTH (β_{24})	**−0.211 ± 0.002*****	**−0.232 ± 0.002*****	**−0.219 ± 0.002*****	**−0.198 ± 0.002*****	**−0.209 ± 0.002*****	**−0.221 ± 0.002*****
SES→COGN (β_{25})	**−0.135 ± 0.002*****	**−0.131 ± 0.002*****	**−0.136 ± 0.002*****	**−0.125 ± 0.002*****	**−0.132 ± 0.002*****	**−0.131 ± 0.002*****
LIFESTYLE→COGN (β_{35})	**+0.015 ± 0.001*****	**+0.016 ± 0.001*****	**−0.023 ± 0.002*****	**−0.029 ± 0.001*****	**−0.014 ± 0.0017*****	**−0.131 ± 0.002*****
Other direct effects of race						
RACE_ETHN→LIFESTYLE (β_{13})	**+0.257 ± 0.009*****	**−0.076 ± 0.009*****	**−0.103 ± 0.007*****	**−0.675 ± 0.009*****	**−0.482 ± 0.007*****	**−0.217 ± 0.006*****
RACE_ETHN→HEALTH(β_{14})	**+0.093 ± 0.006*****	**+0.065 ± 0.006*****	**+0.077 ± 0.006*****	+0.009 ± 0.006	−0.009 ± 0.006	**+0.059 ± 0.006*****
RACE_ETHN→COGN(β_{15})	**+0.523 ± 0.007*****	**+0.528 ± 0.007*****	**+0.524 ± 0.007*****	**+0.507 ± 0.007*****	**+0.521 ± 0.007*****	**+0.520 ± 0.007*****
Selected Indirect effects						
RACE_ETHN → SES → DEMENTIA(β_A)	**+0.077 ± 0.007*****	**+0.075 ± 0.007*****	**+0.076 ± 0.007*****	**+0.068 ± 0.007*****	**+0.073 ± 0.007*****	**+0.071 ± 0.007*****
RACE_ETHN → SES → LIFESTYLE → DEMENTIA(β_B)	−0.002 ± 0.001	+0.0002 ± 0.0003	−0.0002 ± 0.0010	**+0.0081 ± 0.0015*****	**+0.0040 ± 0.0006*****	**+0.0050 ± 0.0006*****
RACE_ETHN → SES → LIFESTYLE → HEALTH → DEMENTIA(β_C)	**+0.0022 ± 0.0001*****	**−0.00076 ± 0.00005*****	**+0.00099 ± 0.00006*****	**+0.0039 ± 0.0002*****	**+0.0023 ± 0.0001*****	**+0.00075 ± 0.0000*****

RACE_ETHN → SES → LIFESTYLE → HEALTH → COGN → DEMENTIA(β_D)	0.0000 ± 0.0000	0.00000 ± 0.00000	+0.0000 ± 0.0000	−0.00004 ± 0.0000***	−0.00002 ± 0.00000***	+0.0000 ± +0.0000**
RACE_ETHN → SES → LIFESTYLE→ COGN → DEMENTIA(β_E)	−0.00043 ± 0.00004***	0.000136 ± 0.000014***	−0.00051 ± 0.00005***	+0.00129 ± 0.00008***	+0.00021 ± 0.0000***	+0.00039 ± 0.00003***
RACE_ETHN → SES → COGN → DEMENTIA(β_F)	+0.0197 ± 0.0009***	0.0191 ± 0.0009***	+0.0198 ± 0.0009***	+0.0181 ± 0.0009***	+0.0192 ± 0.0009***	+0.0188 ± 0.0009***
TOTAL EFFECT OF RACE_ETHN	+0.232 ± 0.078**	+0.232 ± 0.078**	+0.232 ± 0.078**	+0.232 ± 0.078**	+0.232 ± 0.078**	+0.232 ± 0.078**

Abbreviations: AD: Alzheimer's Disease; ALCOHOL: Alcohol consumption z-score; COGN: Poor cognitive performance z-score; DIET: diet quality z-score; HEALTH: Poor cardio-metabolic and general health z-score; NUTR: Nutritional biomarker z-score; PA: Physical Activity z-score; RACE_ETHN: Racial minority status (Non-White vs. White); SES: Socio-economic status z-score; SMOKING: Smoking z-score; SS: Social Support z-score. [a]Values are path coefficients β ± SE or non-linear combinations of path coefficients to compute selected indirect effects. → DEMENTIA associations are interpreted as Log$_e$(HR) of these incident outcomes per unit exposure, as are total effects of RACE_ETHN. $^{*}P < 0.05$ $^{**}P < 0.01$ $^{***}P < 0.001$ for null hypothesis of β = 0.

Supplementary Table 8. Generalized Structural Equations models (GSEM) models for racial/ethnic disparities in all-cause dementia: mediation through SES, alternative lifestyle factors (LIFESTYLE) and health-related factors (HEALTH): The UK Biobank 2006–2021[a].

	LIFESYLTE					
	DIET	PA	SMOKING	ALCOHOL	NUTR	SS
Main pathway						
RACE_ETHN→SES (β_{12})	−0.351 ± 0.006***	−0.350 ± 0.006***	−0.350 ± 0.006***	−0.351 ± 0.006***	−0.351 ± 0.006***	−0.351 ± 0.006***
SES→LIFESTYLE (β_{23})	+0.192 ± 0.003***	−0.059 ± 0.003***	−0.152 ± 0.002***	+0.305 ± 0.002***	+0.102 ± 0.002***	+0.086 ± 0.002***
LIFESTYLE → HEALTH (β_{34})	−0.081 ± 0.001***	−0.091 ± 0.001***	+0.046 ± 0.002***	−0.094 ± 0.001***	−0.168 ± 0.002***	−0.063 ± 0.002***
HEALTH → DEMENTIA(β_{46})	+0.408 ± 0.019***	+0.408 ± 0.019***	+0.404 ± 0.019***	+0.384 ± 0.019***	+0.376 ± 0.020***	+0.394 ± 0.020***
Selected direct effects on final outcomes						
RACE_ETHN→DEMENTIA(β_{16})	+0.092 ± 0.079	+0.104 ± 0.078	+0.101 ± 0.078	+0.048 ± 0.079	+0.050 ± 0.079	+0.067 ± 0.079
SES → DEMENTIA(β_{26})	−0.284 ± 0.020***	−0.278 ± 0.020***	−0.281 ± 0.020***	−0.255 ± 0.020***	−0.272 ± 0.020***	−0.265 ± 0.020***
LIFESTYLE → DEMENTIA(β_{36})	0.032 ± 0.014*	+0.018 ± 0.013	−0.017 ± 0.020	−0.091 ± 0.014***	−0.117 ± 0.017***	−0.184 ± 0.021***
Other effects between endogenous variables						
SES→HEALTH (β_{24})	−0.211 ± 0.002***	−0.232 ± 0.002***	−0.219 ± 0.002***	−0.198 ± 0.002***	+0.102 ± 0.002***	−0.221 ± 0.002***
Other direct effects of race						
RACE_ETHN→LIFESTYLE (β_{13})	+0.257 ± 0.009***	−0.076 ± 0.009***	−0.104 ± 0.007***	−0.675 ± 0.009***	−0.482 ± 0.007***	−0.217 ± 0.006***
RACE_ETHN→HEALTH(β_{14})	+0.093 ± 0.006***	+0.065 ± 0.006***	+0.077 ± 0.006***	+0.009 ± 0.006	−0.009 ± 0.006	+0.059 ± 0.006***
Selected Indirect effects						
RACE_ETHN → SES → DEMENTIA(β_A)	+0.0998 ± 0.0073***	+0.0977 ± 0.0070***	+0.0987 ± 0.0073***	+0.0896 ± 0.0073***	+0.096 ± 0.007***	+0.093 ± 0.007***
RACE_ETHN → SES → LIFESTYLE → DEMENTIA(β_B)	−0.0022 ± 0.0009*	+0.0004 ± 0.0003	−0.0009 ± 0.0010	+0.0097 ± 0.0014***	+0.0042 ± 0.0006***	+0.0055 ± 0.0007***
RACE_ETHN → SES → LIFESTYLE → HEALTH → DEMENTIA(β_C)	+0.0022 ± 0.0001***	−0.00076 ± 0.00005***	+0.000991 ± 0.00006***	+0.0039 ± 0.0002***	+0.0023 ± 0.0001***	+0.00075 ± 0.0000***
TOTAL EFFECT OF RACE_ETHN	+0.232 ± 0.078**	+0.232 ± 0.078**	+0.232 ± 0.078**	+0.232 ± 0.078**	+0.232 ± 0.078**	+0.232 ± 0.078**

Abbreviations: AD: Alzheimer's Disease; ALCOHOL: Alcohol consumption z-score; COGN: Poor cognitive performance z-score; DIET: diet quality z-score; HEALTH: Poor cardio-metabolic and general health z-score; NUTR: Nutritional biomarker z-score; PA: Physical Activity z-score; RACE_ETHN: Racial minority status (Non-White vs. White); SES: Socio-economic status z-score; SMOKING: Smoking z-score; SS: Social Support z-score. [a]Values are path coefficients β ± SE or non-linear combinations of path coefficients to compute selected indirect effects. →DEMENTIA associations are interpreted as Log$_e$(HR) of these incident outcomes per unit exposure, as are total effects of RACE_ETHN. $^{*}P < 0.05$ $^{**}P < 0.01$ $^{***}P < 0.001$ for null hypothesis of β = 0.
